# Lifestyle and Food Habits Impact on Chronic Diseases: Roles of PPARs

**DOI:** 10.3390/ijms20215422

**Published:** 2019-10-31

**Authors:** Michele d’Angelo, Vanessa Castelli, Maria Grazia Tupone, Mariano Catanesi, Andrea Antonosante, Reyes Dominguez-Benot, Rodolfo Ippoliti, Anna Maria Cimini, Elisabetta Benedetti

**Affiliations:** 1 Department of Life, Health and Environmental Sciences, University of L’Aquila, 67100 L’Aquila, Italy; dangelomiche@gmail.com (M.d.); castelli.vane@gmail.com (V.C.); mariagrazia.tupone@gmail.com (M.G.T.); mariano.catanesi86@gmail.com (M.C.); andrea.antonosante@gmail.com (A.A.); rdominguezbenot@unite.it (R.D.-B.); rodolfo.ippoliti@univaq.it (R.I.); annamaria.cimini@univaq.it (A.M.C.); 2 Sbarro Institute for Cancer Research and Molecular Medicine and Center for Biotechnology, Temple University, Philadelphia, PA 19122, USA

**Keywords:** PPARs, inflammation, lifestyle, chronic diseases

## Abstract

Peroxisome proliferator-activated receptors (PPARs) are nuclear receptors that exert important functions in mediating the pleiotropic effects of diverse exogenous factors such as physical exercise and food components. Particularly, PPARs act as transcription factors that control the expression of genes implicated in lipid and glucose metabolism, and cellular proliferation and differentiation. In this review, we aim to summarize the recent advancements reported on the effects of lifestyle and food habits on PPAR transcriptional activity in chronic disease.

## 1. Introduction

Modern lifestyle characterized by unbalanced composition of the diet and poor physical activity, accompanied by the presence of environmental pollutants, has resulted in dramatic increases in the rates of metabolic disease and age-related diseases. These chronic diseases, such as diabetes, cardiovascular disease (CVD), autoimmune diseases, cancers (breast, colorectal, pancreas), and neurodegenerative diseases are all characterized by a chronic sterile systemic low-grade inflammation [1,2,3]. Moreover, these chronic diseases correlate with the metabolic syndrome (MetS), defined by a cluster of interrelated factors: dyslipidemia, hypertension, dysregulated glucose homeostasis, abdominal obesity, and insulin resistance (IR) [4]. Particularly, the obesity and insulin resistance emerge to be the heart of the pathophysiology of the MetS [5]. Different environmental factors of Western lifestyle play a key role in inducing chronic sterile systemic low-grade inflammation and, eventually, the correlated chronic disease. These factors may be divided in the unbalanced composition of the diet [6,7,8] and non-food related factors [9]. Regarding the diet, in Western society there is a consumption of high glycemic index foods (cookies, chocolate, pastries), thus is associated with obesity and IR [10,11,12,13]. This kind of diet increases inflammatory biomarkers [14] and it is related to chronic disease, such as CVD, diabetes, cancer, Alzheimer’s disease [10,15,16,17]. In Western diet there is also an elevated consumption of certain saturated fatty acids (SAFA) [18], and industrially produced trans fatty acids [19,20]. Moreover, in Western diet there is an high ω6/ω3 fatty acid ratio [21,22,23], mostly because of a low intake of long-chain polyunsaturated fatty acids of the ω3 series, such as eicosapentaenoic acid (EPA) and docosahexaenoic acid (DHA) from fish, and alpha-linolenic acid (ALA) from vegetable sources [24]. Among the non-food related factors, it is possible to mention smoking habit, insufficient physical activity [25,26,27,28], and environmental pollution [9] such as exposition to endocrine disruptors [29]. Thus, these kinds of lifestyle and food habits promote a chronic inflammatory status that as mentioned above is characteristic of chronic diseases. Biochemical mediators of lipids are represented by PPARs [30]. This review provides an update of lifestyle and food habits on low grade inflammation in two main chronic diseases, polycystic ovary syndrome (PCOS) and non-alcoholic fatty liver disease (NAFLD), with particular attention on the mechanism that involve the activation of the major metabolic and inflammatory players, the PPARs.

PPARs are ligand-activated transcription factors, belonging to the superfamily of nuclear receptors (NR). PPARs act as lipid sensors; therefore, they have attracted much attention for their ability to improve metabolic syndromes [31]. They take part in nutrient and energy metabolism regulating whole-body energy homeostasis [32,33]. PPARs regulate nutrient metabolism such as lipid, glucose, and cholesterol and sustain the intraorgan metabolic flexibility (Box 1); indeed PPARs play also an important role in regulating the correct inflammation tone [30]. There are three PPARs subtypes: PPARα (NR1C1), PPARβ/δ (NR1C2), and PPARγ (NR1C3), that are highly homologous but differ for tissue distribution and biological functions (Table 1). Fatty acids and their derivatives are the main endogenous agonists of PPARs [34], while among the synthetic ligands there are the main drug utilized for counteracting MetS (Table 1). Their main activity in regulating lipid, glucose metabolism, and inflammation suggests that PPARs are the crossroad of several molecular signaling pathways, implicated in metaflammation onset [35].

## 2. PPARs and Metabolism

All three PPARs are involved in adipose tissue homeostasis. Tissues with high rates of fatty acids catabolism, such as brown adipose tissue (BAT), liver, and skeletal muscles, present high level of PPARα activity. Most PPARα studies have been conducted on the liver [36], in which this nuclear receptor is able to increase the transcription of gene related to the fatty acid transport and catabolism [36,37,38], ketogenesis [37], and gluconeogenesis [39,40]. PPARα in liver is a key factor for the adaptation of fasting and, consequently, energy switch from carbohydrate to fatty acid produced by WAT lipolysis. In the fed state, insulin-dependent PI3K pathway activates rapamycin complex 1 (mTORC1) that in turn suppresses, through nuclear receptor corepressor 1, PPARα activity [41]. PPARα agonist reduces obesity-related metabolic disorders. Experiments conducted on obese mice showed that PPARα agonist treatments improved the obesity condition and glucose homeostasis in terms of glucose intolerance, insulin resistance, and hyperglycemia [42,43]. Goto proposes three options to explain the ability in improving glucose metabolism via adipose tissue [44]. The first option proposes that one of the PPARα capabilities is to increase the expression of a particular hepatokine, the fibroblast growth factor 21 (FGF21) [42], a cytokine able to increase the energy consumptions in white adipose tissue (WAT) via the enhancement of the brown adipose tissue (BAT) activity (generally called “browning”) [45,46]. In fact, the authors showed that fibrate treatment increases the energy consumptions and adipocyte dysfunction and improve glucose homeostasis in WAT of high-fat diet (HFD) wild-type mice, but not in fibroblast growth factor (FGF21)-deficient mice [42]. The second option is the PPARα-mediated enhancing of the production and the release of a particular lipokine, 1-palmitoyl lysophosphatidylcholine, by the liver [47]. This lipokine is able to recover the glucose uptake in insulin-resistant adipocytes, and is an endogenous ligand of PPARα, suggesting a positive feedback loop between PPARα activation and 1-palmitoyl lysophosphatidylcholine production in the liver [47]. Finally, the last option is the improvement of glucose metabolism, via direct action of PPARα on adipose tissue. In fact, transgenic mice that express in adipose tissues constitutive active human PPARα, presented under HFD, recovered insulin sensitivity [48], suggesting an important role of this NR in attenuating obesity-induced insulin resistance in WAT. Two isomeric forms of PPARγ exist, PPARγ1 and PPARγ2; PPARγ1 is most copious in WAT, but it presents also in other tissue (Table 1), while the expression of PPARγ2 is restricted in BAT and WAT [49,50]. Both isoforms are able to induce adipocytes differentiation although PPARγ2 appears more potent in this function [51]. In adipose tissue PPARγ plays key roles in adipocytes differentiation and survival, in the same time, this NR regulates insulin sensitivity and lipogenesis [37,52]. In BAT, the activation of PPARγ triggers the expression of genes linked to thermogenic program, comprising PPARγ coactivator protein 1α (PGC1A) and uncoupling protein 1 (UCP1) [53]. Regarding MetS, PPARγ is the most studied NR since 1995; it was recognized as a molecular target of thiazolidinediones, a class of antidiabetic and insulin-sensitizing drugs [54]. The activation of PPARγ, inducing adipocytes differentiation and strengthening the capacity of lipid accumulation in WAT [50] protects the body from IR and free FA release leading to the attenuation of lipotoxicity. In fact, negative regulation of adipogenic transcription factors, such as PPARγ in adipose tissue, has been demonstrated to cause visceral obesity [55]. Under over-nutrition, the increase of adipose tissue has a protective role in preventing the release of free fatty acids in the systemic circulation. This is possible because in WAT there are stem cells that can differentiate in adipocytes, thus increasing its ability in lipids storage; in this mechanism PPARγ plays an essential role. The fact that fat is not always bad is derived from the evidence that a significant part of obese individuals (healthy obese) do not show dysmetabolism while a significant percentage of lean individuals do [56,57]. Healthy WAT is composed by different adipocytes, showing an increase of hyperplasia and a decrease of hypertrophy; the latter is a definite feature of pathologic obesity [58,59,60,61,62,63]. Recently, it has been demonstrated that the recruit of new adipocytes from PDGFRβ+ pre-adipocytes determines the visceral WAT health in obesity [64]. Notably, in the hypothalamus of HFD rodents, by inhibiting PPARγ in the central nervous system (CNS), the sensitivity of the leptin pathway was improved. Another study demonstrated that transgenic mice knockout for PPARγ in hypothalamic neurons had enhanced energy consumption; on the contrary, food intake and body weight were decreased. In addition, these mice had improved glucose metabolism upon High Fat Diet (HFD) [65]. Thus, PPARγ signaling in the brain influence the energy balance and stimulate the obesity phenotype [66]. Although the same obesogenic effects have been reported for activation of PPARα in the brain, the PPAR β/δ isotype appears to exert opposite role. Mice with PPPAR β/δ deleted showed a strong expression of PPARγ and PPARα in the hypothalamus [67]. Regarding PPAR β/δ, in genetic models, it has been demonstrated that the activation of this NF protects against obesity [68]. Transgenic mice encoding an active form of PPAR β/δ specifically in adipose tissue, fed with a standard chow diet, showed decrease of body weights (20%), of inguinal fat pad masses (40%), and less circulating free FAs and triglycerides compared to control animals [68]. The same mice upon HFD or genetically predisposed to the obesity are protected against weight gain, adipocyte hypertrophy, hypertriglyceridemia, and steatosis [68]. Moreover, an increase of browning was observed in these mice [68]. In opposite, the loss of PPARβ/δ function rendered mice more prone to weight gain and had reduced expression of brown fat UCP1 upon HFD [68]. While PPARα is the most present isoform in the liver, PPARβ/δ isoform is the most expressed in muscle and it is preferentially found in oxidative rather than glycolytic myofibers [69,70,71]. In muscle cells, the activation of PPARβ/δ switches energy production from glycolysis to fatty acid oxidation enhancing muscle endurance [72]. Moreover, the activation of this NF increases the fatty acid uptake and catabolism via oxidation in skeletal muscle cells [73]. PPARβ/δ expression in muscle has several physiological implications such as decreased skeletal muscle fatigability and increased resistance to HFD-induced obesity [71]. Insulin-resistant obese monkeys treated with GW501516; a ligand PPARβ/δ showed an increased serum high-density lipoprotein cholesterol and a decrease of low density lipoprotein, fasting triglycerides, and insulin [74]. The activation of PPARβ/δ, during HFD, increases consumption of lipid in skeletal muscles, avoiding hypertrophy of adipocytes and IR [68,69,75]. Finally, physical exercise and fasting increase the expression of PPARβ/δ in muscles [75,76,77], demonstrating that PPARs act as an interface between lifestyle and health.

Striated muscle plays central roles in MetS, since it is a regulator of total body mass and energy consumption. A surplus of glucose, free fatty acid, and triglycerides concomitant with physical inactivity altered muscular metabolism, that in turn contributes to the onset of obesity and IR [78]. In a healthy-weight individual, skeletal muscles represent ~40% of the total human body mass, and with the cardiac tissue, use almost 30% of the resting energy and nearly 100% of energy utilization during physical exercise [78]. Skeletal muscle is composed of heterogeneous myofibers, slow-, mixed- and fast-twitch, that differ in the composition of contractile protein apparatus and metabolism. In particular, slow-twitch (TypeI) has high oxidative aptitude using fatty acids as substrate for ATP production; mixed oxidative/glycolytic fast-twitch (type IIA) with both phenotype, and type IIB display high strength of contraction but lower oxidative ability doing anaerobic glycolysis [79]. Thus, systemic energy is impacted mostly by fiber type composition [80,81]. Physical activities, especially aerobic exercises, increase the amount of slow fiber type, while the opposite is observed in obesity and diabetes in which there is an enhance of caloric intake without an increase of metabolic demand [82]. Instead, in both diabetic and obese patients, physical activity improve IR and lean mass [83,84]. Similar to adipose tissue, the muscle secretes factors, named myokines, that act in an autocrine and/or paracrine manner [85,86]. Myokines panel production depends on exercise and may modulate, as adipokines, glucose and lipids metabolism [87,88,89]. Among these myokines, myostatin regulate glucose and lipid metabolism, and myostatin-deficient animal are not susceptible to diet-induced obesity [89]. Other myokines involved in systemic metabolism are angiopoietin-like protein 4 (ANGPTL4) [90], irisin, FGF-21, Interleukin-15 (IL-15), [85], meteorin-like protein [91] and Growth differentiation factor 11 (GDF11) [92]. Finally, β-aminoisobutyric acid (BAIBA), belongs to a recent class of factors called “myometabokines,” is able to regulate systemic metabolism crosstalk [86], and to induce browning phenotype in white adipose tissue [93]. These last discoveries highlight the importance of muscle on energy homeostasis and thus the influence of moderate physical activity on human health.

Box 1        **BOX1**

**1. PPARs and Nutrient Metabolism:**

*1.1. Lipid Metabolism*

1.1.1. During the fasting state:

PPARα increases plasma high-density lipoproteins (HDL) levels and reduces low-density lipoproteins (LDL) levels [94,95,96]; it also promotes peroxisomal and mitochondrial oxidation in the protection of the liver from lipotoxicity [97]. Furthermore, PPARα produces and uses ketones during long-term fasting, to nourish extra liver tissues [37].

PPARγ, after being activated, reduces free fatty acids in a systemic way, with the exception of circulating blood and adipose tissue [98].

1.1.2. During normal nutrition:

PPARα produces fatty acids that is used during hunger states, through the coordination of de novo lipogenesis [37].

PPARγ instead is activated during feeding and improves lipid preservation and synthesis, also facilitating the transport of fatty acids to white adipose tissue [37].

PPARβ/δ does not perform a different action based on different nutritional states. It has the ability to inhibit lipogenesis in adipose tissue and improve the catabolism of fatty acids in skeletal muscle [99].

*1.2. Glucose Metabolism*

PPARα decreases glycolysis, improves glycogen synthesis and fatty acid oxidation [100] with consequently inhibiting lipid accumulation.

PPARγ increases gluconeogenesis [101] in the liver and improves glucose-mediated insulin secretion in beta cells in pancreatic Langerhans islands.

PPARβ/δ unlocks its glycolytic action by improving glucose uptake, glycolysis, glycogen storage, and gluconeogenesis reduction [102].

*1.3. Cholesterol Metabolism*

PPARα once activated reduces triglycerides, LDL and increases HDL levels in plasma [103,104]. PPARα also manages to improve cholesterol transport through increased expression of apolipoprotein AI (Apo-AI).

PPARγ agonists, like those of PPARα, regulate the expression of ATP-binding cassette transporter (ABCA1) through increased liver-x-receptor (LXR) expression to increase cholesterol efflux from macrophages via Apo-AI.

PPARβ/δ has effects similar to those of PPARα and PPARγ, namely the increase in plasma HDL levels and the decrease in LDL levels.

PPARβ/δ also reduces the expression of Niemann Pick C1-like 1 (NPC1L1) in the intestine, leading to a reduction in cholesterol adsorption, improving its transintestinal efflux [105].

*1.4. Role of PPARs in Intraorgan Metabolic Flexibility*

1.4.1. PPARs in Heart:

A decrease in PPARα in heart tissue causes decreased antioxidant capacity and structural abnormalities in mitochondria. These two deficits lead to cardiac dysfunction and treatment with the Wy-14643 agonist would improve cardiac function in mouse models [106].

Knockout of PPARγ in mouse model [107] causes cardiac hypertrophy which therefore influences cardiac function and metabolism.

The agonist GW610742 [108] increased PPARβ/δ expression in murine cardiac tissue, improving oxidative and mitochondrial metabolism with a significant decrease in ventricular hypertrophy and a reduction in natriuretic peptide in rats with congestive heart failure.

1.4.2. PPARs in Pancreas:

The role of PPARs in the pancreas has not yet been fully clarified; PPARα and PPARγ levels are known to be very low while PPARβ/δ levels in pancreatic beta cells are highly expressed [109,110].

PPARβ/δ activation improves insulin sensitivity, reduces blood glucose levels, and regulates the expression of genes associated with fatty acid metabolism [111].

PPARγ appears to be implicated [110] in glucose metabolism in pancreatic islets, whereas PPARα overexpression in INS-1 cells reduces lipid accumulation and increases beta-oxidation [31].

1.4.3. PPARs in Skeletal Muscles:

PPARβ/δ maintains energy homeostasis [112] during exercise and in the regulation of mitochondria in skeletal muscle. In fact, PPARβ/δ knockdown leads to a reduction in Peroxisome proliferator-activated receptor gamma coactivator 1-alpha (PGC-1α) levels [113]

PPARγ is involved in the metabolism of glucose in skeletal muscle, improving its absorption.

PPARα instead induces a change in muscle fibers in murine models, when it is over-expressed [114].

1.4.4. PPARs in the Intestine:

The PPARs play an important role in maintaining intestinal microbiota homeostasis.

PPARβ/δ and PPARα are widely expressed in the intestine. Their function is mainly performed in the caecum and distal colon where they can produce short-chain fatty acids [105].

In the small intestine, the PPARα agonist Wy-14643 regulates cholesterol transport and the expression of proteins involved in fatty acid oxidation [115].

The activation of PPARγ has been shown to be useful in improving symptoms because of irritable bowel syndrome [116].

1.4.5. PPARs in Liver:

PPARα is highly expressed in the liver where it plays a key role in fatty acid metabolism, mitochondrial oxidation, and phospholipid remodeling [117].

When PPARβ/δ levels are low or absent, the gene expression associated with the lipoprotein metabolism pathway is diminished, confirming the regulation of triglycerides and cholesterol levels by the PPARβ/δ [118].

The levels of PPARγ in the healthy liver are low [119]. When expressed in the liver of mouse models, it causes liver steatosis. The deletion of PPARα in hepatocytes may induce steatosis [36].

PPARβ/δ is expressed at high levels in hepatocytes, in hepatic macrophages, and in sinusoidal endothelial cells [120].

1.4.6. PPARs in Adipose Tissue

PPARγ plays a key role in adipose tissue. It regulates the differentiation of adipose tissue and participates in the storage and absorption of fatty acids [121].

In fact, the suppression of PPARγ in a fibroblast line inhibits adipose differentiation [51].

Conversely, PPARγ activation induces preservation and transport of fatty acids and activates adipogenesis [122].

PPARα is very well expressed in brown adipose tissue (BAT) but not in white adipose tissue (WAT). PPARα performs its function by regulating thermogenesis and lipid oxidation through interaction with PGC1a in response to adrenergic stimulation of brown adipose tissue [123].

PPARβ/δ instead controls the oxidation of fatty acids in both WAT and BAT [124] tissues and thermogenesis [125] in BAT.

## 3. Systemic Low-Grade Inflammation

Obesity and aging are associated with chronic low-grade inflammation, that in turn is related to chronic diseases [126,127]. Aging is an obesogenic factor, indeed, during aging, a metabolic decline occurs, characterized by altered fat distribution, obesity, and IR [128]. Concomitantly, obesity may worsen age-related diseases [129]. Obesity-associated comorbidities such as hypertension [130], type 2 diabetes [131], and cardiovascular pathologies [132] may finally participate in premature aging and reduced life expectancy. Food intake, metabolism, endocrine system, innate immune responses, and inflammation processes evolve in parallel and concur together in keeping a basal level of inflammation (inflammatory tone), which can be enhanced by the microenvironment (metaflammation) [56], and ageing (inflammaging) [133]. Metaflammation and inflammaging are strongly interconnected; metaflammation might lead and contribute to inflammaging and *vice versa*, that in turn both favor the onset of chronic diseases [133]. In metaflammation and inflammaging, the levels of main circulating pro-inflammatory cytokines, like IL-8, Tumor necrosis factor α (TNFα), IL-6, and IL-1 family, are amplified [134]. Moreover, in both kind of inflammation, the chronic activation of the innate immune system occurs, and macrophages have a central role [133]. The action of this inflammatory cell and their interactions within the stromal components are fundamental for the preservation of tissue homeostasis in metabolic organs, such as liver, brain, pancreas, muscle, and adipose tissue. Indeed, the increase of senescent cells and their accumulation in these tissues, as well the hyperactivation of the innate immune response (Toll-like receptors (TLR) signaling and inflammasome) and the mitochondrial dysfunction have a central role in chronic sterile systemic low grade inflammation [133]. Regarding the food sources, glucose and lipids are able to stimulate the inflammatory response of immune cells [135,136,137]. Recently, Dror and Coll 2017 demonstrated that insulin, a key hormone in glucose metabolism and IL-1β and a master controller of inflammation, support each other in a physiological manner. The authors showed how post-prandial hyperglycemia is associated with a momentary pro-inflammatory response [138,139]. What happens is that in response to glycaemia spike, intraperitoneal macrophages secrete IL-1β which in turn stimulates insulin secretion. IL-1β and insulin increase the glucose uptake in macrophages (preferentially M1), and insulin sustains a pro-inflammatory gene expression profile [138]. Thus, the hyperinsulinemia, driving and supporting the inflammatory state in macrophages, contributes to the induction of chronic low-grade inflammation. Pro-inflammatory responses are also promoted by lipids, cholesterol, mainly in the form of oxidized low-density lipoprotein, is an inducer of a reliable pro-inflammatory response in different kind of cells [140]. SAFA, like palmitate and stearate, are able to activate indirectly TLR 4 and to increase the expression of both TLR2 and TLR4, with consequently production of ROS, activation of NF-κB, and induction of IL-1 synthesis, and MCP-1 release from monocytes [141]. Particularly, fetuin-A, a circulating hepatic glycoprotein, seems to be a transporter of FFAs through the blood stream and an endogenous ligand for TLR4 [142]. Among the long-chain polyunsaturated fatty acids of the ω3 series, docosahexaenoic acid, inhibiting TLR4 and TLR2, exerts its anti-inflammatory role, thus supporting that various lipids from diet affect metaflammation [141,143]. Moreover, the loss of estrogen signaling is one of the key factors of metaflammation onset and the associated immunometabolism alteration [144]. From this point of view the endocrine disruptors present in the environment are becoming an interesting field of study to understand the etiology of some chronic diseases. Finally, alterations in glucose and lipids metabolism, such as the above mentioned, dysregulated glucose homeostasis and dyslipidemia, support cellular senescence in metabolic tissues. Differently to apoptotic cells, senescent cells are not efficiently cleared by the immune system, and their accumulation drives to the progression of chronic and age-related diseases [145]. The senescence-associated secretory phenotype (SASP) has been suggested as a pro-inflammatory activity, contributing to the metaflammation and inflammaging that in turn favor the development of chronic age-related disorders [146].

### 3.1. Adipose Tissue the Core of Metaflammation

Metaflammation is associated with lifestyle and environmental factors, that in some case may cause obesity [56]. Metaflammation originates from white adipose tissue (WAT), an active endocrine organ having a central role in energy balance, glucose homeostasis, and immune functions. Metaflammation may also derive from other metabolic tissues, such as liver, pancreas, and gut [35]. WAT produces different bioactive metabolites and substances such as free fatty acids and bioactive peptides referred to as adipokines [147]. Adipokines include hormones such as leptin, adiponectin, resistin, visfatin and apelin, and chemokines. Among adipokines with inflammatory role there are: TNF-α, monocyte chemotactic protein (MCP-1), plasminogen Activator Inhibitor-1 (PAI-1), IL-1, IL-6, and IL-18; resistin and leptin. While, among insulin-sensitizing adipokines and with anti-inflammatory property: IL-10 and adiponectin. It has been reported that the increased secretion of adipokines and the decreased expression of anti-inflammatory factors, partially cause obesity-related IR [35]. Recently, it has been demonstrated that severe obesity patients undergone a hypocaloric diet and physical exercise program resulted in a metabolic improvement combined with a significant increase of adiponectin levels [148]. Regarding the free fatty acids, in obese condition there are higher levels of circulating saturated fatty acids (SAFAs), proinflammatory lipid compounds that perturb macrophages [149], adipocytes [150], myocytes [151], and hepatocytes [152] inflammation tone, leading to IR [153,154,155]. Omega-6 and saturated fatty acids such as arachidonic acid (AA) and palmitic acid are proinflammatory molecules, while omega-3 fatty acids such as EPA and DHA are anti-inflammatory molecules, for their ability to act as substrates to generate resolvins [156,157], potent mediators that counteract adipose tissue inflammation, decreasing the local adipokines production and monocytes recruitment [158].

WAT is classified in two main types, subcutaneous and visceral WAT, located in the derma and around the internal organs, respectively. As reviewed by Caputo et al. 2017 these kinds of WAT have three important differences. First, the type of adipokines and secretion profile is different; indeed, the visceral WAT has a higher expression and secretion of IL-6 and PAI-1; while leptin and adiponectin are representative adipokines of subcutaneous WAT. Second, visceral and subcutaneous WAT show a different rate of lipolysis and fatty acid mobilization; in particular, the visceral adipose tissue seems to be more vulnerable to the lipolytic activity of catecholamines and less susceptible to the anti-lipolytic activity of insulin. Third, the different bioactive molecules are secreted into the systemic circulation from the subcutaneous WAT, while into the portal system from the visceral WAT. Thus, the latter has a direct impact on hepatic metabolism and consequently on metabolism homeostasis [35]. Moreover, during aging, there is an increase of visceral fat depots with respect to subcutaneous, together with an increase of triglycerides ectopically deposit on muscle, liver, heart, and bone marrow [159]. These alterations are linked to the onset and progression of different age-related diseases [160] supporting the hypothesis that visceral, rather than subcutaneous WAT has a key role in the onset of metaflammation as well as the related metabolic chronic disorders [161]. The obesogen environment acts on WAT inducing the hypertrophy and hyperplasia of the tissue, improving the lipid storage capacity. These events, in particularly in visceral WAT, induce a reduction of WAT vascularization, hypoxia [162,163], and oxidative [164] and endoplasmic reticulum (ER) stress [165], that lead the secretion of a specific panel of free fatty acids and adipokines that recruit inflammatory cells in the tissue [166]. Healthy visceral WAT contains resident macrophages referred to as ATMs, mainly with M2 phenotype (Arg1+, CD206+, CD301+,). ATMs play a key role in the proper maintenance of the tissue inflammatory tone, producing the anti-inflammatory cytokine IL-10. The macrophages recruit in WAT, during the onset of inflammatory process, exhibit mainly a proinflammatory profile (M1 macrophages) [167]. M1 macrophages localize typically in a “crown-like structure,” where the immune cells surround dead or dying adipocytes [168]; this structure is typically used to quantify the levels of inflammation in adipose tissue [169]. Obesogen environment stimulate further inflammation, insulin resistance, and glucose intolerance, also acting on immune cell profile, that undergoes to a massive rearrangement. Recently, it has been demonstrated that a diet enriched with high glycemic index foods, SAFA, and cholesterol support cellular senescence in visceral adipose tissue (visceral WAT) [170]. The senescent cells found in visceral WAT were macrophages [170] and T-cells [35]. Franceschi 2017 clearly states that “the early accumulation of immune senescent cells seems to be a crucial event linking nutritional stress, chronic inflammation, obesity, ageing, and age-related diseases” [146]. Obesity has been proposed as the primary contributing factor in metabolic diseases, however, a significant part of obese individuals (healthy obese) do not show dysmetabolism while a significant percentage of lean individuals do [56,57]. What is emerging is that, in metabolic disease onset not only the amount of adipose tissue plays a key role, but also the remodeling of visceral adipose tissue induced by inflammation and IR. Specifically, these modifications comprise the limitation of visceral WAT to further accumulate lipids, and the presence of senescent cells in visceral WAT that result in increased levels of circulating free fatty acids (FFA) and in a systemic low-grade inflammation.

All three PPAR isotypes have demonstrated anti-inflammatory proprieties [30]; particularly the activation of all PPARs, PPARα [171,172,173,174,175], PPARβ/δ [176,177], and PPARγ [178,179], leads to a decrease of nuclear factor kappa-light-chain-enhancer of activated B cells (NF-κB) activity. However, the anti-inflammatory mechanism is very complex, and takes different forms as extensively and best reviewed by Korbecki et al. 2019 [180]. Moreover, PPARα [181,182] and PPARγ [178,183,184] through their bound to c-Jun, inhibit their ability to bind Activator protein 1 (AP-1) in the promoters of many genes involved in inflammatory process. PPARα and PPARγ disrupt also the activation of signal transducer and activator of transcription proteins (STATs) [185,186].

As explained above, the inflammatory status of visceral adipose tissue plays a pivotal role in the onset of MetS and the related disease. PPARγ plays a fundamental role in adipose tissue differentiation and homeostasis, and it also takes part in modulating inflammatory process in this tissue. In visceral adipose tissue, under physiological conditions, resident immune cells like macrophages and regulatory T-cells generate anti-inflammatory cytokines that maintain glucose homeostasis and the correct inflammatory tone [187]. The onset of metaflammation starts when the immune regulatory network in visceral adipose tissue is disturbed with a decrease in numbers of Tregs and eosinophils and increased recruitment of activated T cells, interferon gamma (IFNγ) -producing natural killer (NK) cells, and inflammatory macrophages [188]. PPARγ activation counteracts the inflammatory process acting on adipocytes and on immune cells. In adipocytes, PPARγ restores the expression and secretion of different anti-inflammatory adipokines [189,190,191]; while on immune cells it has been demonstrated that this NR acts as a negative regulator of macrophages polarization toward M1 phenotype [192], promoting the M2 phenotype [193]. In macrophages, PPARγ induces Arginase 1 (Arg1), a specific M2 marker and sustain β-oxidation and mitochondrial biogenesis [194]; at the same time, it reduces the expression of inflammatory markers [195]. Moreover, the ability of SAFA, like palmitate to activate TLR in macrophages is counteracted by PPARγ [196]; mice lacking PPARγ in myeloid cells, upon HFD, develop obesity and IR [194,197]. In addition, PPARγ activation in Tregs promotes their accumulation in visceral adipose tissue and protection from obesity-induced insulin resistance [187]. More recently, it has been demonstrated also as a key role of PPARγ in dendritic cells c (DC), in fact, in visceral adipose tissue dendritic cells were observed [40,198], although their characterization is not accurate. Under prolonged over-nutrition, these cells process and present antigens to T-cells and induce Th17 responses, while in normal condition they have an anti-inflammatory phenotype and PPARγ plays a fundamental role in maintaining it [199].

### 3.2. Insulin Resistance and Inflammation

Insulin, a peptide hormone secreted by pancreatic β-cells, facilitates the glucose uptake in the cells, thus, it is crucial for maintaining the normal blood glucose level. This hormone has board range of activity; it regulates carbohydrate, protein, and lipid metabolism and also promotes cell division and growth by its mitogenic activities. All metabolic tissues, from liver to brain, are sensible to insulin, therefore, understanding the role of insulin in most of the physiological processes, has significant repercussions for most of the chronic diseases. Insulin uses the adipose tissue, skeletal muscle, and liver as biological buffers against excessive nutrient intake. Since all nutrients are pro-inflammatory [200], insulin exerts a crucial role in preserving the body against their negative effects [201] (Box 2). An excess of nutrients intake may generate an increase of inflammatory tone and compromise the ability of insulin to orchestrate the metabolism. As mentioned above, the unbalanced composition of the diet, such as high glycemic index foods [10] or an elevated consumption of SAFA [202] as well non-food related factors such as insufficient physical activity [203] are associated with IR, and attenuated biological response to normal or elevated insulin (tolerance) [204]. Inflammation and insulin resistance are closely correlated; indeed, important mediators of the inflammatory response, such as NFκB and Jun N-terminal Kinases (JNKs), are able to induce IR, thus establishing a feedback mechanism that feeds the chronic inflammation. TNFα knock-out animal prone to diet-induced obesity (DIO mice) or those that lack leptin (Ob/Ob mice) are able to prevent IR [205]. Target genes of NFκB are involved in IR [206,207]; the phosphorylation of IRS1 in serine-307 by JNKs inhibits the interaction between IRS1 and the insulin receptor, that in turn reduce the insulin receptor inducing IR [208]. Moreover, proinflammatory adipokines produced in obese visceral WAT modulate the activation of JNK and inhibitor of nuclear factor κB (IκB) kinase (IKKβ (implicated in NFκB nuclear translocation), that in turn promotes the onset of IR [35]. The central nervous system (CNS) orchestrates signals for the regulation of food intake and energy expenditure. Particularly, the hypothalamic arcuate nucleus, infundibular nucleus in humans, is a central regulator of feeding behavior, energy, and glucose homeostasis. In this region, the blood–brain barrier results are more permissive, thus resulting as center that communicates peripheral signals into CNS and vice versa. Indeed, the hypothalamus results as the central control point for the development of IR [201]. Satiety signals, such as grelin from the gut, leptin from adipose tissue, and insulin from pancreas, control food intake acting on hypothalamic neurons [209,210]. A diet rich in saturated fats causes inflammation in the hypothalamus, leading to resistance to insulin and leptin as well [211], consequently, the hypothalamus inflammation favor a prominent body weight [212,213,214], setting up a vicious cycle that eventually leads to obesity.

Insulin has a pleiotropic effect on different metabolic tissues; thus, insulin deficiency and insulin resistance have adverse effect on the homeostasis of these tissues. In the adipose tissue, insulin increase fatty acid storage decreasing the activity of hormone-sensitive lipase (HSL) [215]. With the onset of inflammation and insulin resistance in WAT, there is an increase of free fatty acids (FFA) release, which can penetrate into the circulation and are transported by other organs, such as the liver, skeletal muscles, and brain, increasing the insulin resistance in these organs. Among chronic diseases in which IR plays a key role, there are the polycystic ovary syndrome (PCOS) and nonalcoholic fatty liver disease (NAFLD).

Box 2        **BOX2**

**2. The Physiological Roles of Insulin:**

Insulin is an anabolic hormone produced by βcells of the pancreatic islet of Langherans.

Communicating with the liver, muscle, and fat cells, insulin controls the blood glucose levels to be used for energy, and if the body does not request more energy, insulin controls energy conservation, converting glucose in glycogen [216].

*2.1. Insulin in Glucose Metabolism*

Through the insulin-mediated glucose uptake (IMGU) cascade, insulin enhances the glucose absorption from muscle and adipose tissue. Insulin also suppresses glucose production from hepatic cells. Outside of the cell, insulin binds the insulin receptor’s alpha subunit and activates the IMGU cascade, provoking a conformational change in the complex insulin-receptor, inducing the tyrosine kinase phosphorylation of insulin receptor substrate proteins, and the activation of phosphatidylinositol-3-kinase. At this point, intracellular stored Glucose transporter type 4 (GLUT-4) transporter translocates into the skeletal muscle cell’s plasma membrane, and insulin is able to recruit glucose into skeletal muscle cells and converts it into glycogen [217]. In gluconeogenesis, phosphoenolpyruvate carboxylase (PEPc), fructose-1,6-biphosphate (FBP) and glucose-6-phoshate (G6P) are inhibited by insulin. In glycolysis, insulin enhances the expression of glucokinases (GCK) and pyruvate kinase (PKM) [217,218].

*2.2. Insulin in Glycogen Metabolism*

Insulin is able to induce glycogen synthesis in the liver, affecting glycogen metabolism, through the regulation of the key player protein phosphatase I (PPI). PPI can act either by reducing glycogenolysis through the inactivation of phosphorylase kinase and phosphorylase A, or by stimulating glycogenesis inducing the activity of glycogen synthase B. The role of insulin is to increment the specificity of PPI activity, for the control of the synthesis of glycogen from glucose. Furthermore, insulin can affect the expression and activity of several hepatic metabolic enzymes [217,218].

*2.3. Insulin in Lipid Metabolism*

In lipogenesis insulin increases the expression of acetyl-CoA carboxylase (ACC), fatty acid synthase (FAS), and pyruvate dehydrogenase (PDH) [217,218]. Into the adipocytes, glucose is stocked as a lipid, resulting in a major uptake of glucose after fatty acid generation. In this regard insulin up-regulates several lipogenic enzymes expression. Insulin also inhibits lipolysis, through the regulation of dephosphorylation and inhibition of hormone-sensitive lipase [217].

*2.4. Insulin in Protein Metabolism*

Insulin can also regulate protein turnover assessment. In fact, it controls protein synthesis because of the strong expression of insulin in short chain amino acids. Conversely, insulin has the capacity of downregulate hepatic and muscle enzymes, such as ATP-ubiquitin-dependent proteases and ATP-independent lysosomal proteases, affecting protein degradation [216,217,218].

*2.5. Insulin in Inflammation*

In endothelial cells and macrophages, insulin exerts also a potential anti-inflammatory effect. More in depth, inside the endothelial cells insulin can enhance endothelial nitric oxide synthase (eNOS) expression, that releases nitric oxide (NO), resulting in vasodilation. Furthermore, insulin reduces NF-κB present in the endothelial cells. NF-κB up-regulates E-selectin and Intercellular Adhesion Molecule 1 (ICAM-1) adhesion molecules expression, recently found associated with the development of atherosclerotic arterial plaques.

Insulin is also able to reduce the generation of reactive oxygen species (ROS) and O2 radicals. Specifically, insulin reduces the expression of NADPH oxidase, through the suppression of p47phox. NADPH oxidase is responsible of oxygen radicals generation, with consequent activation of the inhibitor of NF-κB kinase beta (IKKB). IKKB phosphorylates IκB, resulting in its degradation. At this point, NF-κB is released and it can translocate into the macrophage’s nucleus, where it promotes several pro-inflammatory proteins gene transcription, such as monocyte chemoattractant protein (MCP-1), interleukin-6 (IL-6), interleukin-8 (IL-8), tumor necrosis factor-alpha (TNF-α), and matrix metalloproteinases (MMPs) [218,219].

## 4. Chronic Diseases, from Lifestyle to PPARs

### 4.1. Polycystic Ovary Syndrome

Polycystic ovary syndrome (PCOS) is the most widespread endocrine disorder affecting 8% to 13% of women [220,221,222]. These women typically present hyperandrogenism, amenorrhea, and polycystic ovaries [223,224]. Patients with PCOS show obesity (visceral phenotype), lipid disorders, IR, compensatory hyperinsulinemia, and they have a higher risk of type 2 diabetes mellitus, metabolic syndrome, cardiovascular complications, reproductive disorders (best review by [225]). Regarding the IR, the hyperinsulinemia-associated increases pituitary luteinizing hormone (LH) secretion and ovary theca cells androgen production; contextually it suppresses the sex hormone-binding globulin (SHBG) production in the liver leading to an enhance of free androgens in the blood that in turn further worsens the insulin resistance [226]. Different studies reported that, about 40–60% of PCOS patients are obese or overweight [227,228], and it has been described an increment in visceral adipose tissue even in lean women with PCOS [229,230]. The pro-inflammatory state of obesity promotes IR and atherogenesis [231], thus in PCOS, the development of obesity induces a decline in insulin sensitivity [226]. Moreover, in PCOS women an imbalance between pro-inflammatory and anti-inflammatory mediators is associated with a systemic low-grade inflammation [232,233,234]; the lipotoxicity could be the key of PCOS pathogenesis [235]. The etiology of this chronic disease is still not fully understood [236]; it is probably due to a combination of genetic and environmental factors. Genetic factors have great importance in the pathophysiology of PCOS [237,238]. The genes involved are associated to cellular metabolism, chronic inflammation, cell proliferation, reproductive hormones [239,240,241,242,243,244]. In this regard, different clinical studies on the incidence of the PPARγ Pro12Ala polymorphism in PCOS patients were reported [245,246,247,248,249,250]; this polymorphism is associated with a lower degree of IR, an increased insulin clearance and a reduced risk of diabetes [250]. However, further clinical studies on the incidence of Pro12Ala in PCOS are necessary. Therapeutic modalities, which are able to reduce IR and lipotoxicity, typically result in improvements in ovarian functions. PCOS patients have low expression of PPARγ in skeletal muscle that it was associated with IR. Treatments with PPAR agonists ameliorated muscle IR and increased the expression of PPARγ with overall increase in mitochondrial biogenesis and function [251]. Moreover, enhancing triglyceride accumulation in adipose tissue, PPARγ agonists are able to diminish lipotoxicity [252,253]. The environmental factors exert an essential function in the development of PCOS [228,254]; among them diet composition, physical activity, and environmental pollution need to be included [228,255,256]. In a recent study, conducted on female nonhuman primates, it has been demonstrated that the combination of mild hyperandrogenemia and Western-style diet prompts the development of visceral WAT dysfunction [257]. Diets containing unsaturated fatty acids (omega-3) and diets with low glycemic index may reduce the risk of metabolic features seen in PCOS patients [258,259] while diets enriched of SAFA and foods with a high glycemic index exert opposite effects [228]. Physical exercise exerts positive effects in the PCOS women’ health, including reduced IR and improved reproductive biomarkers (antral follicle count, serum levels of sex steroids, gonadotropins and anti-Müllerian hormone (AMH) [255]. Lifestyle intervention is able to recover levels of FSH, SHBG, androstenedione, total testosterone, free androgen index, and Ferriman-Gallwey score in PCOS patients [260]. Thus, a correct diet in PCOS patients should include minimal amounts of SAFA with normal quantities of saturated fatty acids with one double bond and omega 3. Furthermore, adequate intake of fiber-rich food and carbohydrate sources with low glycemic index is highly proposed. This kind of diet accompanied with physical activity is proposed as first-line management in an international evidence-based guideline on PCOS [261]. However, lifestyle modifications having long-term effects and sustainability are not so simple, thus pharmacotherapy is often required, such as metformin and troglitazone (TZD) [262]. Particularly, thiazolidinediones, insulin sensitizer drugs, are PPARγ agonists that directly target lipotoxicity and androgen production [263], both of which are dysfunctional in PCOS. Interestingly, other studies found that PPARγ agonist modulates steroid genesis. In a randomized clinical trial, including normoinsulinemic and non-obese PCOS patients, it was found that rosiglitazone, a PPARγ agonist, significantly reduced testosterone levels, without changing insulin levels [264]. Thus, PPARγ agonists may restore the normal androgenic response to insulin. In addition, in vitro studies reported that activation of PPARγ with troglitazone decreased LH stimulation of androgen synthesis in thecal tissue reducing steroid 17 alpha-hydroxylase/17,20 lyase (P450c17) action, a key enzyme for androgen synthesis [265]. In adrenocortical cell line another agonist of PPARγ, pioglitazone, reduced the overexpression of P450c17 [266]. The effects of the PPARγ ligands in PCOS women, especially on IR and steroid genesis indicate a possible role of PPARγ in the pathophysiology of PCOS. However, several studies disclosed that the use of PPARγ agonists have numerous side effects, including cardiac complications, bone resorption, and bladder cancer [267,268]. For this reason, for PCOS patients, new therapeutic approaches able to ameliorate lipotoxicity and/or IR are necessary.

### 4.2. Non-Alcoholic Fatty Liver Disease (NAFLD)

The term nonalcoholic fatty liver disease (NAFLD) comprises a range of liver conditions characterized by an abnormal accumulation of fat in the liver (hepatosteatosis). The incidence of NAFLD is increasing around the world, especially in Western countries [269], thus lifestyle and food habits have a great impact on the onset of chronic diseases [270]. Non-alcoholic fatty liver disease comprises two kind of forms: non-alcoholic fatty liver (NAFL) and non-alcoholic steatohepatitis (NASH) [271,272]. NAFL is characterized by hepatosteatosis and with long-term prognosis, and can develop into NASH, a more aggressive form of liver disease, characterized by liver inflammation and that it may evolve in advanced cirrhosis and even hepatocellular carcinoma [272,273]. The hallmarks of NAFLD are obesity and IR, both of them are crucial for the progression into NASH [274]. The underlying mechanism beside the shift of NAFL toward NASH is complex and multi-factorial [275]. The two-hit theory is the most accredited theory regarding NAFLD pathogenesis [276]. This theory supposes that initially obesity and IR lead to lipid accumulation in hepatocytes [276,277] and subsequently, oxidative stress appears, which in turn, precedes and causes mitochondrial dysfunctions [278,279]. Regarding the first part, the liver steatosis derived diminishes hepatocytes fatty acid (FA) oxidation [36,280] and enhanced (FA) uptake [281,282]. Regarding the FA uptake in the liver, it is mediated by fatty acid transporters such as, cluster of differentiation 36 (CD36) and Fatty Acid Transport Proteins (FATPs) [281,282]. Moreover, it has been shown that polymorphism in genes for lipid transport and lipid metabolism are related to the development of NAFLD [283]. Free FAs derive principally from adipose tissue (59%), dietary, and de novo lipogenesis [35,284]. Particularly, the increase of de novo lipogenesis in liver comes first from steatosis and it is due to a certain extent to the IR of the muscle, which leads to increased flux of glucose toward the liver [35,285]. The hepatocytes with increased de novo lipogenic rate undergo a phenotypic shift, characterized by higher transcription of adipogenic genes, including sterol regulatory element-binding proteins (SREBPs), adipose differentiation- related protein (ADRP), and PPARγ [35,286,287]. One of the unresolved questions in NAFLD is what factors could be the driving forces to the inflammatory disease phenotype. It has been demonstrated that choosing a healthy lifestyle leads to weight loss and improvement of liver fibrosis; overall counteracting NASH evolution [288]. NAFLD patients usually have unhealthy habits such as sedentary lifestyles and unhealthy diet [289]. Although in Western countries, NAFLD is associated with obesity and IR; it has been demonstrated that, particularly in Asiatic population, NAFLD can develop in absence of insulin resistance and with low BMI. Moreover, in urban regions of Asia and Africa, lack of physical activity and the globalization of Western diet has led to an increase in the occurrence of NAFLD [290,291]. On the contrary in the rural areas of these continents, despite the Western diet, there is a lower incidence of NAFLD due to the physical activity [290,291]. The lifestyle-based treatments comprise caloric restriction, improved diet composition, exercise increment, stress reduction, and improved sleep [292]. Caloric restriction together with change in diet composition improve the management of NAFLD [292]. A diet enriched in carbohydrates leads to increased insulin and triglyceride concentrations in the blood, lipogenesis, and IR of the liver in NAFLD patients [293]. Another important diet factor in NAFLD progression is the glycemic index [294]. Diets improved in low glycemic index food help to reduce the overall fat mass, increasing lipid utilization, and increasing satiety [295,296] and makes the liver healthy by decreasing fatty acid accumulation and glycogen storage in liver [294]. Physical exercise exerts positive effects in the NAFLD patients’ health, especially when combined with a correct diet, making improvement in liver enzymes and hepatic histology [297]. Aerobic exercises increase circulating concentrations endocannabinoids, as well as related lipids oleoylethanolamide (OEA), palmitoylethanolamide (PEA), *N*-docsahexaenoylethanolamine (DHEA), and 2-oleoylglycerol (2-OG) [298], particularly OEA is an endogenous ligand of PPARα at is known to ameliorate NAFLD condition [299]. Polyunsaturated fatty acids (PUFAs), particularly DHA and EPA have protective effects in NAFLD patients [300]; on the contrary a diet enriched of SAFA, trans-fatty acids, and cholesterol is associated with NAFLD development or progression [301,302,303]. Studies conducted on mice reported that this kind of diet induced systemic inflammation and liver injuries; moreover, a study on humans indicated that cholesterol is linked to hepatic inflammation and fibrosis [304]. Under physiological conditions, PUFA or their oxidized metabolites control hepatic lipid metabolism acting as PPARα ligands and thus controlling the expression of genes encoding for proteins involved in fatty acids oxidation and transport [305]; in addition, PUFA acts as down-regulators of the lipogenic transcription factors [306,307,308]. Thus, PUFA depletion in hepatic tissue of obese NAFLD patients might trigger FA and Triacylglycerol (TAG) synthesis respect to FA oxidation, inducing hepatic steatosis. These findings are according to nutritional disequilibrium with a high ω6/ω3 fatty acid ratio diet in mice; this kind of diet induced hepatic SREBP and increased lipogenesis, with substantial decrease of FA oxidation and steatosis development [309]. As explained in the section of PPARs and inflammation, both NF-κB and AP-1 may form heterodimers with PPAR-α, determining the development of the transcriptionally inactive complexes p65-PPARα and c-Jun-PPARα [310]. PPARα downregulation in liver has also dramatic consequence in term of inflammation, thus this NR may exert pivotal pathogenic roles, taking into account their significance in inflammation and energy homeostasis. PPARα agonists, i.e., fibrates utilized to reduce steatosis and inflammation in human steatohepatitis, showed weak effectiveness because of the different response of human respects to rodents [35]. Regarding thiazolidinediones, their use in NAFLD is controversial since they can improve IR and decrease lipotoxicity in obese/NAFLD patients, supporting the transcription of Sterol regulatory element-binding protein 1 c (SREBP1c) in the liver, thus enhancing the maintenance of steatosis and production of TGs by liver [311]. Recently, more attention was focused on the possible use of natural compound such as PUFAn3 [312] and oleoylethanolamide [299] natural ligands of PPARα.

## 5. Conclusions

It is becoming noticeable that the primary cause of most Western chronic diseases, with systemic low-grade inflammation as the common denominator, is not following a correct lifestyle and improper food habits. Ruiz-Núñez and Colleagues 2013 [313] deduce as human predisposition to develop IR depends on the rapid brain growth in the past millennium. During this period, the interaction between our immune system and metabolism was strongly conserved, indeed, with the advent of the agricultural and industrial revolutions, leads to chronic inflammation. Lifestyle modifications in Western countries are necessary especially in the first years of life. However, during pathology onset, since improving lifestyle is not that easy, pharmacotherapy is required. PPARs represent the interface between environment metabolism and immune system; moreover, their presence in all metabolic tissues suggests that they play an important role in regulating the fine crosstalk between them. Thus, these receptors are targets for the therapy of metabolic syndrome and the low-grade inflammatory state. Because of the collateral effects induced by fenofibrates and TZDs, new therapeutic approaches are necessary in order to obtain new PPARs ligands characterized by minor negative effects and increased positive effects. Recently, more attention is pointed toward the possible use of natural compounds, such as PUFAn3, oleoylethanolamide and β-aminoisobutyric acid, natural and endogenous ligands of PPARs. Finally, the development of PPARα/γ/δ pan-agonists or PPARα/γ dual agonist [314] could be a potential therapy for a concomitant pharmacological activity on carbohydrate and lipid metabolism.

## Figures and Tables

**Table 1:** PPARs tissue distribution and biological functions.

Isoforms	Tissues Distribution	Target Genes	Functions	Synthetic Ligands	Natural Ligands
PPARγ	White and brown adipose tissue, the large intestine, skeletal muscle, spleen, pancreas, and brain.	*aP2*, *FATP*, *FAT/CD36*.	Regulation of adipogenesis, energy balance, lipogenesis, gluconeogenesis, lipid storage, glucose uptake, metabolism uptake and differentiation.	Rosiglitazone, Pioglitazone, Troglitazone, T3D-959, DBZ.	9-HODE, 13-HODE, 15d-PGJ2, EPA.
PPARα	Liver, heart, skeletal muscle, intestinal mucosa, white and brown adipose tissue, pancreas, and brain.	*Acyl-CoA oxidase*, *Thiolase*, *Apolipoprotein A-I*, *Apolipoprotein A-II*, *CYP8B1*, *FATP*, *FAT/CD36*, and *Lipoprotein lipase*.	Fatty acid metabolism, inflammation, thermogenesis, ketogenesis, glucose uptake, fatty acid oxidation and lipid storage.	Wy-14643, GW-2331, GW-9578, K-877 Fibrates.	Palmitic acid, Oleic acid, Linoleic acid, Arachidonic acid, DHA, oleoylethanolamide.
PPARβ/δ	Liver, intestine, kidney, abdominal white and brown adipose tissue, skeletal muscle, heart, pancreas, and brain.	Genes involved in lipid uptake, metabolism, and efflux, *Lpin2*, *St3gal5*.	Fatty acid oxidation, fatty acid metabolism, regulates blood cholesterol, glucose uptake, glucose utilization, insulin secretion, ketogenesis and inflammation.	L-796449, L-783483, GW-2433, MBX-8025, T3D-959, GW501516, GW610742.	Dihomo-γ-linolenic acid, Arachidonic acid, Methyl palmitate, 2-bromopalmitic acid, prostacyclin I2, 4-HNE.

## References

[1] Reaven G.M. (2005). The Insulin Resistance Syndrome: Definition and Dietary Approaches to Treatment. Annu. Rev. Nutr..

[2] Dandona P., Aljada A., Chaudhuri A., Mohanty P., Garg R. (2005). Metabolic Syndrome: A Comprehensive Perspective Based on Interactions between Obesity, Diabetes, and Inflammation. Circulation.

[3] Libby P. (2002). Inflammation in atherosclerosis. Nature.

[4] Kassi E., Pervanidou P., Kaltsas G., Chrousos G. (2011). Metabolic syndrome: Definitions and controversies. BMC Med..

[5] Cornier M.-A., Dabelea D., Hernandez T.L., Lindstrom R.C., Steig A.J., Stob N.R., Van Pelt R.E., Wang H., Eckel R.H. (2008). The Metabolic Syndrome. Endocr. Rev..

[6] Galland L. (2010). Diet and Inflammation. Nutr. Clin. Pract..

[7] Anand P., Kunnumakkara A.B., Kunnumakara A.B., Sundaram C., Harikumar K.B., Tharakan S.T., Lai O.S., Sung B., Aggarwal B.B. (2008). Cancer is a preventable disease that requires major lifestyle changes. Pharm. Res..

[8] Egger G., Dixon J. (2010). Inflammatory effects of nutritional stimuli: Further support for the need for a big picture approach to tackling obesity and chronic disease. Obes. Rev..

[9] Egger G., Dixon J. (2011). Non-nutrient causes of low-grade, systemic inflammation: Support for a ‘canary in the mineshaft’ view of obesity in chronic disease: Inflammation and obesity. Obes. Rev..

[10] Blaak E.E. (2016). Carbohydrate quantity and quality and cardio-metabolic risk. Curr. Opin. Clin. Nutr. Metab. Care.

[11] Kaartinen N.E., Knekt P., Kanerva N., Valsta L.M., Eriksson J.G., Rissanen H., Jääskeläinen T., Männistö S. (2016). Dietary carbohydrate quantity and quality in relation to obesity: A pooled analysis of three Finnish population-based studies. Scand. J. Public Health.

[12] Cornejo-Monthedoro A., Negreiros-Sánchez I., Del Águila C., Ysla-Marquillo M., Mayta-Tristán P. (2017). Association between dietary glycemic load and metabolic syndrome in obese children and adolescents. Arch. Argent. Pediatr..

[13] Silva K.C., Neri Nobre L., Emanuelle de Castro Ferreira Vicente S., Lopes Moreira L., do Carmo Lessa A., Lamounier J.A. (2016). Influence of glycemic index and glycemic load of the diet on the risk of overweight and adiposity in childhood. Rev. Paul. Pediatr..

[14] Milajerdi A., Saneei P., Larijani B., Esmaillzadeh A. (2018). The effect of dietary glycemic index and glycemic load on inflammatory biomarkers: A systematic review and meta-analysis of randomized clinical trials. Am. J. Clin. Nutr..

[15] Bell S.J., Sears B. (2003). Low-glycemic-load diets: Impact on obesity and chronic diseases. Crit. Rev. Food Sci. Nutr..

[16] Taylor M.K., Sullivan D.K., Swerdlow R.H., Vidoni E.D., Morris J.K., Mahnken J.D., Burns J.M. (2017). A high-glycemic diet is associated with cerebral amyloid burden in cognitively normal older adults. Am. J. Clin. Nutr..

[17] Hu J., La Vecchia C., Augustin L.S., Negri E., de Groh M., Morrison H., Mery L. (2013). Canadian Cancer Registries Epidemiology Research Group Glycemic index, glycemic load and cancer risk. Ann. Oncol..

[18] Jiménez-Gómez Y., López-Miranda J., Blanco-Colio L.M., Marín C., Pérez-Martínez P., Ruano J., Paniagua J.A., Rodríguez F., Egido J., Pérez-Jiménez F. (2009). Olive oil and walnut breakfasts reduce the postprandial inflammatory response in mononuclear cells compared with a butter breakfast in healthy men. Atherosclerosis.

[19] Mozaffarian D. (2006). Trans fatty acids—Effects on systemic inflammation and endothelial function. Atheroscler. Suppl..

[20] Mozaffarian D., Aro A., Willett W.C. (2009). Health effects of trans-fatty acids: Experimental and observational evidence. Eur. J. Clin. Nutr..

[21] Serhan C.N., Chiang N. (2008). Endogenous pro-resolving and anti-inflammatory lipid mediators: A new pharmacologic genus. Br. J. Pharmacol..

[22] Simopoulos A.P. (2008). The Importance of the Omega-6/Omega-3 Fatty Acid Ratio in Cardiovascular Disease and Other Chronic Diseases. Exp. Biol. Med..

[23] Calder P.C. (2006). n−3 Polyunsaturated fatty acids, inflammation, and inflammatory diseases. Am. J. Clin. Nutr..

[24] He K., Liu K., Daviglus M.L., Jenny N.S., Mayer-Davis E., Jiang R., Steffen L., Siscovick D., Tsai M., Herrington D. (2009). Associations of Dietary Long-Chain n-3 Polyunsaturated Fatty Acids and Fish With Biomarkers of Inflammation and Endothelial Activation (from the Multi-Ethnic Study of Atherosclerosis [MESA]). Am. J. Cardiol..

[25] Huffman K.M., Samsa G.P., Slentz C.A., Duscha B.D., Johnson J.L., Bales C.W., Tanner C.J., Houmard J.A., Kraus W.E. (2006). Response of high-sensitivity C-reactive protein to exercise training in an at-risk population. Am. Heart J..

[26] Petersen A.M.W., Pedersen B.K. (2005). The anti-inflammatory effect of exercise. J. Appl. Physiol..

[27] Roubenoff R. (2008). Molecular Basis of Inflammation: Relationships between Catabolic Cytokines, Hormones, Energy Balance, and Muscle. J. Parenter. Enter. Nutr..

[28] Handschin C., Spiegelman B.M. (2008). The role of exercise and PGC1α in inflammation and chronic disease. Nature.

[29] Petrakis D., Vassilopoulou L., Mamoulakis C., Psycharakis C., Anifantaki A., Sifakis S., Docea A., Tsiaoussis J., Makrigiannakis A., Tsatsakis A. (2017). Endocrine Disruptors Leading to Obesity and Related Diseases. Int. J. Environ. Res. Public Health.

[30] Wahli W., Michalik L. (2012). PPARs at the crossroads of lipid signaling and inflammation. Trends Endocrinol. Metab..

[31] Hong F., Pan S., Guo Y., Xu P., Zhai Y. (2019). PPARs as Nuclear Receptors for Nutrient and Energy Metabolism. Molecules.

[32] Schupp M., Lazar M.A. (2010). Endogenous Ligands for Nuclear Receptors: Digging Deeper. J. Biol. Chem..

[33] Bensinger S.J., Tontonoz P. (2008). Integration of metabolism and inflammation by lipid-activated nuclear receptors. Nature.

[34] Woller A., Duez H., Staels B., Lefranc M. (2016). A Mathematical Model of the Liver Circadian Clock Linking Feeding and Fasting Cycles to Clock Function. Cell Rep..

[35] Caputo T., Gilardi F., Desvergne B. (2017). From chronic overnutrition to metaflammation and insulin resistance: Adipose tissue and liver contributions. FEBS Lett..

[36] Montagner A., Polizzi A., Fouché E., Ducheix S., Lippi Y., Lasserre F., Barquissau V., Régnier M., Lukowicz C., Benhamed F. (2016). Liver PPARα is crucial for whole-body fatty acid homeostasis and is protective against NAFLD. Gut.

[37] Dubois V., Eeckhoute J., Lefebvre P., Staels B. (2017). Distinct but complementary contributions of PPAR isotypes to energy homeostasis. J. Clin. Investig..

[38] Lefebvre P. (2006). Sorting out the roles of PPAR in energy metabolism and vascular homeostasis. J. Clin. Investig..

[39] Jitrapakdee S., Slawik M., Medina-Gomez G., Campbell M., Wallace J.C., Sethi J.K., O’Rahilly S., Vidal-Puig A.J. (2005). The Peroxisome Proliferator-activated Receptor-γ Regulates Murine Pyruvate Carboxylase Gene Expression in Vivo and in Vitro. J. Biol. Chem..

[40] Patsouris D., Mandard S., Voshol P.J., Escher P., Tan N.S., Havekes L.M., Koenig W., März W., Tafuri S., Wahli W. (2004). PPARα governs glycerol metabolism. J. Clin. Investig..

[41] Sengupta S., Peterson T.R., Laplante M., Oh S., Sabatini D.M. (2010). mTORC1 controls fasting-induced ketogenesis and its modulation by ageing. Nature.

[42] Goto T., Hirata M., Aoki Y., Iwase M., Takahashi H., Kim M., Li Y., Jheng H.-F., Nomura W., Takahashi N. (2017). The hepatokine FGF21 is crucial for peroxisome proliferator-activated receptor-α agonist-induced amelioration of metabolic disorders in obese mice. J. Biol. Chem..

[43] Goto T., Lee J.-Y., Teraminami A., Kim Y.-I., Hirai S., Uemura T., Inoue H., Takahashi N., Kawada T. (2011). Activation of peroxisome proliferator-activated receptor-alpha stimulates both differentiation and fatty acid oxidation in adipocytes. J. Lipid Res..

[44] Goto T. (2019). A review of the studies on food-derived factors which regulate energy metabolism via the modulation of lipid-sensing nuclear receptors. Biosci. Biotechnol. Biochem..

[45] Coskun T., Bina H.A., Schneider M.A., Dunbar J.D., Hu C.C., Chen Y., Moller D.E., Kharitonenkov A. (2008). Fibroblast Growth Factor 21 Corrects Obesity in Mice. Endocrinology.

[46] Fisher F.M., Kleiner S., Douris N., Fox E.C., Mepani R.J., Verdeguer F., Wu J., Kharitonenkov A., Flier J.S., Maratos-Flier E. (2012). FGF21 regulates PGC-1 and browning of white adipose tissues in adaptive thermogenesis. Genes Dev..

[47] Takahashi H., Goto T., Yamazaki Y., Kamakari K., Hirata M., Suzuki H., Shibata D., Nakata R., Inoue H., Takahashi N. (2015). Metabolomics reveal 1-palmitoyl lysophosphatidylcholine production by peroxisome proliferator-activated receptor α. J. Lipid Res..

[48] Takahashi H., Sanada K., Nagai H., Li Y., Aoki Y., Ara T., Seno S., Matsuda H., Yu R., Kawada T. (2017). Over-expression of PPARα in obese mice adipose tissue improves insulin sensitivity. Biochem. Biophys. Res. Commun..

[49] Tontonoz P., Spiegelman B.M. (2008). Fat and Beyond: The Diverse Biology of PPARγ. Annu. Rev. Biochem..

[50] Lehrke M., Lazar M.A. (2005). The Many Faces of PPARγ. Cell.

[51] Rosen E.D. (2002). C/EBPalpha induces adipogenesis through PPARgamma: A unified pathway. Genes Dev..

[52] Lefterova M.I., Haakonsson A.K., Lazar M.A., Mandrup S. (2014). PPARγ and the global map of adipogenesis and beyond. Trends Endocrinol. Metab..

[53] Inagaki T., Sakai J., Kajimura S. (2016). Transcriptional and epigenetic control of brown and beige adipose cell fate and function. Nat. Rev. Mol. Cell Biol..

[54] Lehmann J.M., Moore L.B., Smith-Oliver T.A., Wilkison W.O., Willson T.M., Kliewer S.A. (1995). An Antidiabetic Thiazolidinedione Is a High Affinity Ligand for Peroxisome Proliferator-activated Receptor γ (PPARγ). J. Biol. Chem..

[55] Sharma A.M., Staels B. (2007). Peroxisome Proliferator-Activated Receptor γ and Adipose Tissue—Understanding Obesity-Related Changes in Regulation of Lipid and Glucose Metabolism. J. Clin. Endocrinol. Metab..

[56] Egger G., Dixon J. (2009). Obesity and chronic disease: Always offender or often just accomplice?. Br. J. Nutr..

[57] Wildman R.P. (2008). The Obese without Cardiometabolic Risk Factor Clustering and the Normal Weight with Cardiometabolic Risk Factor Clustering: Prevalence and Correlates of 2 Phenotypes Among the US Population (NHANES 1999-2004). Arch. Intern. Med..

[58] Hoffstedt J., Arner E., Wahrenberg H., Andersson D.P., Qvisth V., Löfgren P., Rydén M., Thörne A., Wirén M., Palmér M. (2010). Regional impact of adipose tissue morphology on the metabolic profile in morbid obesity. Diabetologia.

[59] Gustafson B., Gogg S., Hedjazifar S., Jenndahl L., Hammarstedt A., Smith U. (2009). Inflammation and impaired adipogenesis in hypertrophic obesity in man. Am. J. Physiol.-Endocrinol. Metab..

[60] Lee M.-J., Wu Y., Fried S.K. (2010). Adipose tissue remodeling in pathophysiology of obesity. Curr. Opin. Clin. Nutr. Metab. Care.

[61] Sun K., Kusminski C.M., Scherer P.E. (2011). Adipose tissue remodeling and obesity. J. Clin. Investig..

[62] Gustafson B., Hammarstedt A., Hedjazifar S., Smith U. (2013). Restricted Adipogenesis in Hypertrophic Obesity: The Role of WISP2, WNT, and BMP4. Diabetes.

[63] Klöting N., Blüher M. (2014). Adipocyte dysfunction, inflammation and metabolic syndrome. Rev. Endocr. Metab. Disord..

[64] Shao M., Vishvanath L., Busbuso N.C., Hepler C., Shan B., Sharma A.X., Chen S., Yu X., An Y.A., Zhu Y. (2018). De novo adipocyte differentiation from Pdgfrβ+ preadipocytes protects against pathologic visceral adipose expansion in obesity. Nat. Commun..

[65] Stump M., Guo D.-F., Lu K.-T., Mukohda M., Liu X., Rahmouni K., Sigmund C.D. (2016). Effect of selective expression of dominant-negative PPARγ in pro-opiomelanocortin neurons on the control of energy balance. Physiol. Genom..

[66] Long L., Toda C., Jeong J.K., Horvath T.L., Diano S. (2014). PPARγ ablation sensitizes proopiomelanocortin neurons to leptin during high-fat feeding. J. Clin. Investig..

[67] Kocalis H.E., Turney M.K., Printz R.L., Laryea G.N., Muglia L.J., Davies S.S., Stanwood G.D., McGuinness O.P., Niswender K.D. (2012). Neuron-Specific Deletion of Peroxisome Proliferator-Activated Receptor Delta (PPARδ) in Mice Leads to Increased Susceptibility to Diet-Induced Obesity. PLoS ONE.

[68] Wang H., Liu H., Liu R.M. (2003). Gender difference in glutathione metabolism during aging in mice. Exp. Gerontol..

[69] Tanaka T., Yamamoto J., Iwasaki S., Asaba H., Hamura H., Ikeda Y., Watanabe M., Magoori K., Ioka R.X., Tachibana K. (2003). Activation of peroxisome proliferator-activated receptor induces fatty acid -oxidation in skeletal muscle and attenuates metabolic syndrome. Proc. Natl. Acad. Sci. USA.

[70] Braissant O., Foufelle F., Scotto C., Dauça M., Wahli W. (1996). Differential expression of peroxisome proliferator-activated receptors (PPARs): Tissue distribution of PPAR-alpha, -beta, and -gamma in the adult rat. Endocrinology.

[71] Wang Y.-X., Zhang C.-L., Yu R.T., Cho H.K., Nelson M.C., Bayuga-Ocampo C.R., Ham J., Kang H., Evans R.M. (2004). Regulation of Muscle Fiber Type and Running Endurance by PPARδ. PLoS Biol..

[72] Fan W., Waizenegger W., Lin C.S., Sorrentino V., He M.-X., Wall C.E., Li H., Liddle C., Yu R.T., Atkins A.R. (2017). PPARδ Promotes Running Endurance by Preserving Glucose. Cell Metab..

[73] Holst D., Luquet S., Nogueira V., Kristiansen K., Leverve X., Grimaldi P.A. (2003). Nutritional regulation and role of peroxisome proliferator-activated receptor δ in fatty acid catabolism in skeletal muscle. Biochim. Biophys. Acta (BBA)-Mol. Cell Biol. Lipids.

[74] Oliver W.R., Shenk J.L., Snaith M.R., Russell C.S., Plunket K.D., Bodkin N.L., Lewis M.C., Winegar D.A., Sznaidman M.L., Lambert M.H. (2001). A selective peroxisome proliferator-activated receptor agonist promotes reverse cholesterol transport. Proc. Natl. Acad. Sci. USA.

[75] Luquet S., Lopez-Soriano J., Holst D., Fredenrich A., Melki J., Rassoulzadegan M., Grimaldi P.A. (2003). Peroxisome proliferator-activated receptor δ controls muscle development and oxidative capability. FASEB J..

[76] Watt M.J., Southgate R.J., Holmes A.G., Febbraio M.A. (2004). Suppression of plasma free fatty acids upregulates peroxisome proliferator-activated receptor (PPAR) α and δ and PPAR coactivator 1α in human skeletal muscle, but not lipid regulatory genes. J. Mol. Endocrinol..

[77] Mahoney D.J., Parise G., Melov S., Safdar A., Tarnopolsky M.A. (2005). Analysis of global mRNA expression in human skeletal muscle during recovery from endurance exercise. FASEB J..

[78] Baskin K.K., Winders B.R., Olson E.N. (2015). Muscle as a “Mediator” of Systemic Metabolism. Cell Metab..

[79] Pette D., Staron R.S. (2000). Myosin isoforms, muscle fiber types, and transitions. Microsc. Res. Tech..

[80] Bassel-Duby R., Olson E.N. (2006). Signaling Pathways in Skeletal Muscle Remodeling. Annu. Rev. Biochem..

[81] Schiaffino S., Reggiani C. (2011). Fiber types in mammalian skeletal muscles. Physiol. Rev..

[82] Mootha V.K., Lindgren C.M., Eriksson K.-F., Subramanian A., Sihag S., Lehar J., Puigserver P., Carlsson E., Ridderstråle M., Laurila E. (2003). PGC-1α-responsive genes involved in oxidative phosphorylation are coordinately downregulated in human diabetes. Nat. Genet..

[83] Zanuso S., Jimenez A., Pugliese G., Corigliano G., Balducci S. (2010). Exercise for the management of type 2 diabetes: A review of the evidence. Acta Diabetol..

[84] Willis L.H., Slentz C.A., Bateman L.A., Shields A.T., Piner L.W., Bales C.W., Houmard J.A., Kraus W.E. (2012). Effects of aerobic and/or resistance training on body mass and fat mass in overweight or obese adults. J. Appl. Physiol..

[85] Pedersen B.K., Febbraio M.A. (2012). Muscles, exercise and obesity: Skeletal muscle as a secretory organ. Nat. Rev. Endocrinol..

[86] Weigert C., Lehmann R., Hartwig S., Lehr S. (2014). The secretome of the working human skeletal muscle--a promising opportunity to combat the metabolic disaster?. Proteom. Clin. Appl..

[87] Pedersen B.K., Steensberg A., Fischer C., Keller C., Keller P., Plomgaard P., Wolsk-Petersen E., Febbraio M. (2004). The metabolic role of IL-6 produced during exercise: Is IL-6 an exercise factor?. Proc. Nutr. Soc..

[88] Muñoz-Cánoves P., Scheele C., Pedersen B.K., Serrano A.L. (2013). Interleukin-6 myokine signaling in skeletal muscle: A double-edged sword?. FEBS J..

[89] Huang Z., Chen X., Chen D. (2011). Myostatin: A novel insight into its role in metabolism, signal pathways, and expression regulation. Cell. Signal..

[90] Mattijssen F., Kersten S. (2012). Regulation of triglyceride metabolism by Angiopoietin-like proteins. Biochim. Biophys. Acta.

[91] Rao R.R., Long J.Z., White J.P., Svensson K.J., Lou J., Lokurkar I., Jedrychowski M.P., Ruas J.L., Wrann C.D., Lo J.C. (2014). Meteorin-like is a hormone that regulates immune-adipose interactions to increase beige fat thermogenesis. Cell.

[92] Sinha M., Jang Y.C., Oh J., Khong D., Wu E.Y., Manohar R., Miller C., Regalado S.G., Loffredo F.S., Pancoast J.R. (2014). Restoring systemic GDF11 levels reverses age-related dysfunction in mouse skeletal muscle. Science.

[93] Roberts L.D., Boström P., O’Sullivan J.F., Schinzel R.T., Lewis G.D., Dejam A., Lee Y.-K., Palma M.J., Calhoun S., Georgiadi A. (2014). β-Aminoisobutyric acid induces browning of white fat and hepatic β-oxidation and is inversely correlated with cardiometabolic risk factors. Cell Metab..

[94] Schoonjans K., Peinado-Onsurbe J., Lefebvre A.M., Heyman R.A., Briggs M., Deeb S., Staels B., Auwerx J. (1996). PPARalpha and PPARgamma activators direct a distinct tissue-specific transcriptional response via a PPRE in the lipoprotein lipase gene. EMBO J..

[95] Vu-Dac N., Gervois P., Jakel H., Nowak M., Bauge E., Dehondt H., Staels B., Pennacchio L.A., Rubin E.M., Fruchart-Najib J. (2003). Apolipoprotein A5, a crucial determinant of plasma triglyceride levels, is highly responsive to peroxisome proliferator-activated receptor alpha activators. J. Biol. Chem..

[96] Berthou L., Duverger N., Emmanuel F., Langouët S., Auwerx J., Guillouzo A., Fruchart J.C., Rubin E., Denèfle P., Staels B. (1996). Opposite regulation of human versus mouse apolipoprotein A-I by fibrates in human apolipoprotein A-I transgenic mice. J. Clin. Investig..

[97] Pawlak M., Lefebvre P., Staels B. (2015). Molecular mechanism of PPARα action and its impact on lipid metabolism, inflammation and fibrosis in non-alcoholic fatty liver disease. J. Hepatol..

[98] Gross B., Pawlak M., Lefebvre P., Staels B. (2017). PPARs in obesity-induced T2DM, dyslipidaemia and NAFLD. Nat. Rev. Endocrinol..

[99] Derosa G., Sahebkar A., Maffioli P. (2018). The role of various peroxisome proliferator-activated receptors and their ligands in clinical practice. J. Cell. Physiol..

[100] Peeters A., Baes M. (2010). Role of PPAR in Hepatic Carbohydrate Metabolism. PPAR Res..

[101] Ahmadian M., Suh J.M., Hah N., Liddle C., Atkins A.R., Downes M., Evans R.M. (2013). PPARγ signaling and metabolism: The good, the bad and the future. Nat. Med..

[102] Liu S., Hatano B., Zhao M., Yen C.-C., Kang K., Reilly S.M., Gangl M.R., Gorgun C., Balschi J.A., Ntambi J.M. (2011). Role of Peroxisome Proliferator-activated Receptor δ/β in Hepatic Metabolic Regulation. J. Biol. Chem..

[103] Rubins H.B., Robins S.J., Collins D. (1996). The veterans affairs high-density lipoprotein intervention trial: Baseline characteristics of normocholesterolemic men with coronary artery disease and low levels of high-density lipoprotein cholesterol. Am. J. Cardiol..

[104] Manninen V., Tenkanen L., Koskinen P., Huttunen J.K., Mänttäri M., Heinonen O.P., Frick M.H. (1992). Joint effects of serum triglyceride and LDL cholesterol and HDL cholesterol concentrations on coronary heart disease risk in the Helsinki Heart Study. Implications for treatment. Circulation.

[105] Vrins C.L.J., van der Velde A.E., van den Oever K., Levels J.H.M., Huet S., Oude Elferink R.P.J., Kuipers F., Groen A.K. (2009). Peroxisome proliferator-activated receptor delta activation leads to increased transintestinal cholesterol efflux. J. Lipid Res..

[106] Lee T.-W., Bai K.-J., Lee T.-I., Chao T.-F., Kao Y.-H., Chen Y.-J. (2017). PPARs modulate cardiac metabolism and mitochondrial function in diabetes. J. Biomed. Sci..

[107] Duan S.Z., Ivashchenko C.Y., Russell M.W., Milstone D.S., Mortensen R.M. (2005). Cardiomyocyte-Specific Knockout and Agonist of Peroxisome Proliferator–Activated Receptor-γ Both Induce Cardiac Hypertrophy in Mice. Circ. Res..

[108] Vázquez-Carrera M. (2016). Unraveling the Effects of PPARβ/δ on Insulin Resistance and Cardiovascular Disease. Trends Endocrinol. Metab..

[109] Wan J., Jiang L., Lü Q., Ke L., Li X., Tong N. (2010). Activation of PPARδ up-regulates fatty acid oxidation and energy uncoupling genes of mitochondria and reduces palmitate-induced apoptosis in pancreatic β-cells. Biochem. Biophys. Res. Commun..

[110] Li L., Li T., Zhang Y., Pan Z., Wu B., Huang X., Zhang Y., Mei Y., Ge L., Shen G. (2015). Peroxisome proliferator-activated receptorβ/δ activation is essential for modulating p-Foxo1/Foxo1 status in functional insulin-positive cell differentiation. Cell Death Dis..

[111] Cohen G., Riahi Y., Shamni O., Guichardant M., Chatgilialoglu C., Ferreri C., Kaiser N., Sasson S. (2011). Role of Lipid Peroxidation and PPAR-δ in Amplifying Glucose-Stimulated Insulin Secretion. Diabetes.

[112] Koh J.-H., Hancock C.R., Terada S., Higashida K., Holloszy J.O., Han D.-H. (2017). PPARβ Is Essential for Maintaining Normal Levels of PGC-1α and Mitochondria and for the Increase in Muscle Mitochondria Induced by Exercise. Cell Metab..

[113] Schnuck J.K., Sunderland K.L., Gannon N.P., Kuennen M.R., Vaughan R.A. (2016). Leucine stimulates PPARβ/δ-dependent mitochondrial biogenesis and oxidative metabolism with enhanced GLUT4 content and glucose uptake in myotubes. Biochimie.

[114] Finck B.N., Bernal-Mizrachi C., Han D.H., Coleman T., Sambandam N., LaRiviere L.L., Holloszy J.O., Semenkovich C.F., Kelly D.P. (2005). A potential link between muscle peroxisome proliferator- activated receptor-α signaling and obesity-related diabetes. Cell Metab..

[115] Karimian Azari E., Leitner C., Jaggi T., Langhans W., Mansouri A. (2013). Possible Role of Intestinal Fatty Acid Oxidation in the Eating-Inhibitory Effect of the PPAR-α Agonist Wy-14643 in High-Fat Diet Fed Rats. PLoS ONE.

[116] Nozu T., Miyagishi S., Nozu R., Takakusaki K., Okumura T. (2019). Pioglitazone improves visceral sensation and colonic permeability in a rat model of irritable bowel syndrome. J. Pharmacol. Sci..

[117] Régnier M., Polizzi A., Lippi Y., Fouché E., Michel G., Lukowicz C., Smati S., Marrot A., Lasserre F., Naylies C. (2018). Insights into the role of hepatocyte PPARα activity in response to fasting. Mol. Cell. Endocrinol..

[118] Sanderson L.M., Boekschoten M.V., Desvergne B., Müller M., Kersten S. (2010). Transcriptional profiling reveals divergent roles of PPARα and PPARβ/δ in regulation of gene expression in mouse liver. Physiol. Genom..

[119] Morán-Salvador E., López-Parra M., García-Alonso V., Titos E., Martínez-Clemente M., González-Périz A., López-Vicario C., Barak Y., Arroyo V., Clària J. (2011). Role for PPARγ in obesity-induced hepatic steatosis as determined by hepatocyte- and macrophage-specific conditional knockouts. FASEB J..

[120] Hoekstra M., Kruijt J.K., Van Eck M., van Berkel T.J.C. (2003). Specific Gene Expression of ATP-binding Cassette Transporters and Nuclear Hormone Receptors in Rat Liver Parenchymal, Endothelial, and Kupffer Cells. J. Biol. Chem..

[121] Tontonoz P., Hu E., Graves R.A., Budavari A.I., Spiegelman B.M. (1994). mPPAR gamma 2: Tissue-specific regulator of an adipocyte enhancer. Genes Dev..

[122] Xiao L., Zhang J., Li H., Liu J., He L., Zhang J., Zhai Y. (2010). Inhibition of adipocyte differentiation and adipogenesis by the traditional Chinese herb *Sibiraea Angustata*. Exp. Biol. Med..

[123] Teng C.T., Li Y., Stockton P., Foley J. (2011). Fasting Induces the Expression of PGC-1α and ERR Isoforms in the Outer Stripe of the Outer Medulla (OSOM) of the Mouse Kidney. PLoS ONE.

[124] Dressel U., Allen T.L., Pippal J.B., Rohde P.R., Lau P., Muscat G.E.O. (2003). The Peroxisome Proliferator-Activated Receptor β/δ Agonist, GW501516, Regulates the Expression of Genes Involved in Lipid Catabolism and Energy Uncoupling in Skeletal Muscle Cells. Mol. Endocrinol..

[125] Wang Y.-X., Lee C.-H., Tiep S., Yu R.T., Ham J., Kang H., Evans R.M. (2003). Peroxisome-Proliferator-Activated Receptor δ Activates Fat Metabolism to Prevent Obesity. Cell.

[126] Lumeng C.N., Saltiel A.R. (2011). Inflammatory links between obesity and metabolic disease. J. Clin. Investig..

[127] Woods J.A., Wilund K.R., Martin S.A., Kistler B.M. (2012). Exercise, inflammation and aging. Aging Dis..

[128] Gabriely I., Ma X.H., Yang X.M., Rossetti L., Barzilai N. (2002). Leptin Resistance During Aging Is Independent of Fat Mass. Diabetes.

[129] Villareal D.T., Apovian C.M., Kushner R.F., Klein S. (2005). Obesity in Older Adults: Technical Review and Position Statement of the American Society for Nutrition and NAASO, the Obesity Society. Obes. Res..

[130] Kotsis V., Stabouli S., Papakatsika S., Rizos Z., Parati G. (2010). Mechanisms of obesity-induced hypertension. Hypertens. Res..

[131] Eckel R.H., Kahn S.E., Ferrannini E., Goldfine A.B., Nathan D.M., Schwartz M.W., Smith R.J., Smith S.R. (2011). Obesity and Type 2 Diabetes: What Can Be Unified and What Needs to Be Individualized?. Diabetes Care.

[132] Poirier P., Giles T.D., Bray G.A., Hong Y., Stern J.S., Pi-Sunyer F.X., Eckel R.H. (2006). Obesity and Cardiovascular Disease: Pathophysiology, Evaluation, and Effect of Weight Loss: An Update of the 1997 American Heart Association Scientific Statement on Obesity and Heart Disease From the Obesity Committee of the Council on Nutrition, Physical Activity, and Metabolism. Circulation.

[133] Franceschi C., Garagnani P., Parini P., Giuliani C., Santoro A. (2018). Inflammaging: A new immune–metabolic viewpoint for age-related diseases. Nat. Rev. Endocrinol..

[134] Prattichizzo F., De Nigris V., La Sala L., Procopio A.D., Olivieri F., Ceriello A. (2016). “Inflammaging” as a Druggable Target: A Senescence-Associated Secretory Phenotype—Centered View of Type 2 Diabetes. Oxidative Med. Cell. Longev..

[135] Johnson A.R., Justin Milner J., Makowski L. (2012). The inflammation highway: Metabolism accelerates inflammatory traffic in obesity. Immunol. Rev..

[136] Tanaka M., Masuda S., Matsuo Y., Sasaki Y., Yamakage H., Muranaka K., Wada H., Hasegawa K., Tsukahara T., Shimatsu A. (2016). Hyperglycemia and Inflammatory Property of Circulating Monocytes are Associated with Inflammatory Property of Carotid Plaques in Patients Undergoing Carotid Endarterectomy. J. Atheroscler. Thromb..

[137] Liu X., Madhankumar A.B., Miller P.A., Duck K.A., Hafenstein S., Rizk E., Slagle-Webb B., Sheehan J.M., Connor J.R., Yang Q.X. (2016). MRI contrast agent for targeting glioma: Interleukin-13 labeled liposome encapsulating gadolinium-DTPA. Neuro-Oncology.

[138] Dror E., Dalmas E., Meier D.T., Wueest S., Thévenet J., Thienel C., Timper K., Nordmann T.M., Traub S., Schulze F. (2017). Postprandial macrophage-derived IL-1β stimulates insulin, and both synergistically promote glucose disposal and inflammation. Nat. Immunol..

[139] Esposito K., Nappo F., Marfella R., Giugliano G., Giugliano F., Ciotola M., Quagliaro L., Ceriello A., Giugliano D. (2002). Inflammatory Cytokine Concentrations Are Acutely Increased by Hyperglycemia in Humans: Role of Oxidative Stress. Circulation.

[140] Duewell P., Kono H., Rayner K.J., Sirois C.M., Vladimer G., Bauernfeind F.G., Abela G.S., Franchi L., Nuñez G., Schnurr M. (2010). NLRP3 inflammasomes are required for atherogenesis and activated by cholesterol crystals. Nature.

[141] Dasu M.R., Ramirez S., Isseroff R.R. (2012). Toll-like receptors and diabetes: A therapeutic perspective. Clin. Sci..

[142] Pal D., Dasgupta S., Kundu R., Maitra S., Das G., Mukhopadhyay S., Ray S., Majumdar S.S., Bhattacharya S. (2012). Fetuin-A acts as an endogenous ligand of TLR4 to promote lipid-induced insulin resistance. Nat. Med..

[143] Lee J.Y., Sohn K.H., Rhee S.H., Hwang D. (2001). Saturated Fatty Acids, but Not Unsaturated Fatty Acids, Induce the Expression of Cyclooxygenase-2 Mediated through Toll-like Receptor 4. J. Biol. Chem..

[144] Drew B.G., Hamidi H., Zhou Z., Villanueva C.J., Krum S.A., Calkin A.C., Parks B.W., Ribas V., Kalajian N.Y., Phun J. (2015). Estrogen receptor (ER)α-regulated lipocalin 2 expression in adipose tissue links obesity with breast cancer progression. J. Biol. Chem..

[145] Kirkland J.L., Tchkonia T. (2017). Cellular Senescence: A Translational Perspective. EBioMedicine.

[146] Franceschi C. (2017). Obesity in geroscience—Is cellular senescence the culprit?. Nat. Rev. Endocrinol..

[147] Jung U., Choi M.-S. (2014). Obesity and Its Metabolic Complications: The Role of Adipokines and the Relationship between Obesity, Inflammation, Insulin Resistance, Dyslipidemia and Nonalcoholic Fatty Liver Disease. Int. J. Mol. Sci..

[148] Corbi G., Polito R., Monaco M.L., Cacciatore F., Scioli M., Ferrara N., Daniele A., Nigro E. (2019). Adiponectin Expression and Genotypes in Italian People with Severe Obesity Undergone a Hypocaloric Diet and Physical Exercise Program. Nutrients.

[149] Nguyen M.T.A., Favelyukis S., Nguyen A.-K., Reichart D., Scott P.A., Jenn A., Liu-Bryan R., Glass C.K., Neels J.G., Olefsky J.M. (2007). A Subpopulation of Macrophages Infiltrates Hypertrophic Adipose Tissue and Is Activated by Free Fatty Acids via Toll-like Receptors 2 and 4 and JNK-dependent Pathways. J. Biol. Chem..

[150] Nguyen M.T.A., Satoh H., Favelyukis S., Babendure J.L., Imamura T., Sbodio J.I., Zalevsky J., Dahiyat B.I., Chi N.-W., Olefsky J.M. (2005). JNK and Tumor Necrosis Factor-α Mediate Free Fatty Acid-induced Insulin Resistance in 3T3-L1 Adipocytes. J. Biol. Chem..

[151] Yu C., Chen Y., Cline G.W., Zhang D., Zong H., Wang Y., Bergeron R., Kim J.K., Cushman S.W., Cooney G.J. (2002). Mechanism by Which Fatty Acids Inhibit Insulin Activation of Insulin Receptor Substrate-1 (IRS-1)-associated Phosphatidylinositol 3-Kinase Activity in Muscle. J. Biol. Chem..

[152] Hwang D., Rhee S.H. (1999). Receptor-mediated signaling pathways: Potential targets of modulation by dietary fatty acids. Am. J. Clin. Nutr..

[153] Glass C.K., Olefsky J.M. (2012). Inflammation and lipid signaling in the etiology of insulin resistance. Cell Metab..

[154] Park E., Wong V., Guan X., Oprescu A.I., Giacca A. (2007). Salicylate prevents hepatic insulin resistance caused by short-term elevation of free fatty acids in vivo. J. Endocrinol..

[155] Summers S. (2006). Ceramides in insulin resistance and lipotoxicity. Prog. Lipid Res..

[156] Serhan C.N., Brain S.D., Buckley C.D., Gilroy D.W., Haslett C., O’Neill L.A.J., Perretti M., Rossi A.G., Wallace J.L. (2007). Resolution of inflammation: State of the art, definitions and terms. FASEB J..

[157] Serhan C.N. (2007). Resolution Phase of Inflammation: Novel Endogenous Anti-Inflammatory and Proresolving Lipid Mediators and Pathways. Annu. Rev. Immunol..

[158] Clària J., Dalli J., Yacoubian S., Gao F., Serhan C.N. (2012). Resolvin D1 and Resolvin D2 Govern Local Inflammatory Tone in Obese Fat. J. Immunol..

[159] Kuk J.L., Saunders T.J., Davidson L.E., Ross R. (2009). Age-related changes in total and regional fat distribution. Ageing Res. Rev..

[160] Mau T., Yung R. (2018). Adipose tissue inflammation in aging. Exp. Gerontol..

[161] Fox C.S., Massaro J.M., Hoffmann U., Pou K.M., Maurovich-Horvat P., Liu C.-Y., Vasan R.S., Murabito J.M., Meigs J.B., Cupples L.A. (2007). Abdominal visceral and subcutaneous adipose tissue compartments: Association with metabolic risk factors in the Framingham Heart Study. Circulation.

[162] Hausman D.B., DiGirolamo M., Bartness T.J., Hausman G.J., Martin R.J. (2001). The biology of white adipocyte proliferation. Obes. Rev..

[163] Lee Y.S., Kim J., Osborne O., Oh D.Y., Sasik R., Schenk S., Chen A., Chung H., Murphy A., Watkins S.M. (2014). Increased Adipocyte O2 Consumption Triggers HIF-1α, Causing Inflammation and Insulin Resistance in Obesity. Cell.

[164] Furukawa S., Fujita T., Shimabukuro M., Iwaki M., Yamada Y., Nakajima Y., Nakayama O., Makishima M., Matsuda M., Shimomura I. (2004). Increased oxidative stress in obesity and its impact on metabolic syndrome. J. Clin. Investig..

[165] Ozcan U. (2004). Endoplasmic Reticulum Stress Links Obesity, Insulin Action, and Type 2 Diabetes. Science.

[166] Weisberg S.P., McCann D., Desai M., Rosenbaum M., Leibel R.L., Ferrante A.W. (2003). Obesity is associated with macrophage accumulation in adipose tissue. J. Clin. Investig..

[167] Lumeng C.N., Bodzin J.L., Saltiel A.R. (2007). Obesity induces a phenotypic switch in adipose tissue macrophage polarization. J. Clin. Investig..

[168] Murano I., Barbatelli G., Parisani V., Latini C., Muzzonigro G., Castellucci M., Cinti S. (2008). Dead adipocytes, detected as crown-like structures, are prevalent in visceral fat depots of genetically obese mice. J. Lipid Res..

[169] Zamarron B.F., Mergian T.A., Cho K.W., Martinez-Santibanez G., Luan D., Singer K., DelProposto J.L., Geletka L.M., Muir L.A., Lumeng C.N. (2017). Macrophage Proliferation Sustains Adipose Tissue Inflammation in Formerly Obese Mice. Diabetes.

[170] Schafer M.J., White T.A., Evans G., Tonne J.M., Verzosa G.C., Stout M.B., Mazula D.L., Palmer A.K., Baker D.J., Jensen M.D. (2016). Exercise Prevents Diet-Induced Cellular Senescence in Adipose Tissue. Diabetes.

[171] Huang D., Zhao Q., Liu H., Guo Y., Xu H. (2016). PPAR-α Agonist WY-14643 Inhibits LPS-Induced Inflammation in Synovial Fibroblasts via NF-κB Pathway. J. Mol. Neurosci..

[172] Staels B., Koenig W., Habib A., Merval R., Lebret M., Torra I.P., Delerive P., Fadel A., Chinetti G., Fruchart J.C. (1998). Activation of human aortic smooth-muscle cells is inhibited by PPARalpha but not by PPARgamma activators. Nature.

[173] Rival Y., Benéteau N., Taillandier T., Pezet M., Dupont-Passelaigue E., Patoiseau J.F., Junquéro D., Colpaert F.C., Delhon A. (2002). PPARalpha and PPARdelta activators inhibit cytokine-induced nuclear translocation of NF-kappaB and expression of VCAM-1 in EAhy926 endothelial cells. Eur. J. Pharmacol..

[174] Dubrac S., Stoitzner P., Pirkebner D., Elentner A., Schoonjans K., Auwerx J., Saeland S., Hengster P., Fritsch P., Romani N. (2007). Peroxisome proliferator-activated receptor-alpha activation inhibits Langerhans cell function. J. Immunol..

[175] Ramanan S., Kooshki M., Zhao W., Hsu F.-C., Robbins M.E. (2008). PPARalpha ligands inhibit radiation-induced microglial inflammatory responses by negatively regulating NF-kappaB and AP-1 pathways. Free Radic. Biol. Med..

[176] Zingarelli B., Piraino G., Hake P.W., O’Connor M., Denenberg A., Fan H., Cook J.A. (2010). Peroxisome proliferator-activated receptor {delta} regulates inflammation via NF-{kappa}B signaling in polymicrobial sepsis. Am. J. Pathol..

[177] Barroso E., Eyre E., Palomer X., Vázquez-Carrera M. (2011). The peroxisome proliferator-activated receptor β/δ (PPARβ/δ) agonist GW501516 prevents TNF-α-induced NF-κB activation in human HaCaT cells by reducing p65 acetylation through AMPK and SIRT1. Biochem. Pharmacol..

[178] Han S., Inoue H., Flowers L.C., Sidell N. (2003). Control of COX-2 gene expression through peroxisome proliferator-activated receptor gamma in human cervical cancer cells. Clin. Cancer Res..

[179] He X., Liu W., Shi M., Yang Z., Zhang X., Gong P. (2017). Docosahexaenoic acid attenuates LPS-stimulated inflammatory response by regulating the PPARγ/NF-κB pathways in primary bovine mammary epithelial cells. Res. Vet. Sci..

[180] Korbecki J., Bobiński R., Dutka M. (2019). Self-regulation of the inflammatory response by peroxisome proliferator-activated receptors. Inflamm. Res..

[181] Delerive P., De Bosscher K., Besnard S., Vanden Berghe W., Peters J.M., Gonzalez F.J., Fruchart J.C., Tedgui A., Haegeman G., Staels B. (1999). Peroxisome proliferator-activated receptor alpha negatively regulates the vascular inflammatory gene response by negative cross-talk with transcription factors NF-kappaB and AP-1. J. Biol. Chem..

[182] Liang C.-J., Tseng C.-P., Yang C.-M., Ma Y.-H. (2011). 20-Hydroxyeicosatetraenoic acid inhibits ATP-induced COX-2 expression via peroxisome proliferator activator receptor-α in vascular smooth muscle cells: 20-HETE inhibits COX-2 expression. Br. J. Pharmacol..

[183] Subbaramaiah K., Lin D.T., Hart J.C., Dannenberg A.J. (2001). Peroxisome Proliferator-activated Receptor γ Ligands Suppress the Transcriptional Activation of Cyclooxygenase-2: EVIDENCE FOR INVOLVEMENT OF ACTIVATOR PROTEIN-1 AND CREB-BINDING PROTEIN/p300. J. Biol. Chem..

[184] Khandoudi N., Delerive P., Berrebi-Bertrand I., Buckingham R.E., Staels B., Bril A. (2002). Rosiglitazone, a Peroxisome Proliferator-Activated Receptor-, Inhibits the Jun NH2-Terminal Kinase/Activating Protein 1 Pathway and Protects the Heart From Ischemia/Reperfusion Injury. Diabetes.

[185] Ji H.-G., Piao J.-Y., Kim S.-J., Kim D.-H., Lee H.-N., Na H.-K., Surh Y.-J. (2016). Docosahexaenoic acid inhibits Helicobacter pylori-induced STAT3 phosphorylation through activation of PPARγ. Mol. Nutr. Food Res..

[186] Yu J.H., Kim K.H., Kim H. (2008). SOCS 3 and PPAR-gamma ligands inhibit the expression of IL-6 and TGF-beta1 by regulating JAK2/STAT3 signaling in pancreas. Int. J. Biochem. Cell Biol..

[187] Cipolletta D., Feuerer M., Li A., Kamei N., Lee J., Shoelson S.E., Benoist C., Mathis D. (2012). PPAR-γ is a major driver of the accumulation and phenotype of adipose tissue Treg cells. Nature.

[188] Schipper H.S., Prakken B., Kalkhoven E., Boes M. (2012). Adipose tissue-resident immune cells: Key players in immunometabolism. Trends Endocrinol. Metab..

[189] Steppan C.M., Bailey S.T., Bhat S., Brown E.J., Banerjee R.R., Wright C.M., Patel H.R., Ahima R.S., Lazar M.A. (2001). The hormone resistin links obesity to diabetes. Nature.

[190] Peraldi P., Xu M., Spiegelman B.M. (1997). Thiazolidinediones block tumor necrosis factor-alpha-induced inhibition of insulin signaling. J. Clin. Investig..

[191] Hammarstedt A., Andersson C.X., Rotter Sopasakis V., Smith U. (2005). The effect of PPARγ ligands on the adipose tissue in insulin resistance. Prostaglandins Leukot. Essent. Fat. Acids.

[192] Chawla A. (2010). Control of Macrophage Activation and Function by PPARs. Circ. Res..

[193] Huang J.T., Welch J.S., Ricote M., Binder C.J., Willson T.M., Kelly C., Witztum J.L., Funk C.D., Conrad D., Glass C.K. (1999). Interleukin-4-dependent production of PPAR-γ ligands in macrophages by 12/15-lipoxygenase. Nature.

[194] Odegaard J.I., Ricardo-Gonzalez R.R., Goforth M.H., Morel C.R., Subramanian V., Mukundan L., Red Eagle A., Vats D., Brombacher F., Ferrante A.W. (2007). Macrophage-specific PPARgamma controls alternative activation and improves insulin resistance. Nature.

[195] Xu H., Barnes G.T., Yang Q., Tan G., Yang D., Chou C.J., Sole J., Nichols A., Ross J.S., Tartaglia L.A. (2003). Chronic inflammation in fat plays a crucial role in the development of obesity-related insulin resistance. J. Clin. Investig..

[196] Kratz M., Coats B.R., Hisert K.B., Hagman D., Mutskov V., Peris E., Schoenfelt K.Q., Kuzma J.N., Larson I., Billing P.S. (2014). Metabolic dysfunction drives a mechanistically distinct proinflammatory phenotype in adipose tissue macrophages. Cell Metab..

[197] Hevener A.L., Olefsky J.M., Reichart D., Nguyen M.T.A., Bandyopadyhay G., Leung H.-Y., Watt M.J., Benner C., Febbraio M.A., Nguyen A.-K. (2007). Macrophage PPAR gamma is required for normal skeletal muscle and hepatic insulin sensitivity and full antidiabetic effects of thiazolidinediones. J. Clin. Investig..

[198] Bertola A., Ciucci T., Rousseau D., Bourlier V., Duffaut C., Bonnafous S., Blin-Wakkach C., Anty R., Iannelli A., Gugenheim J. (2012). Identification of adipose tissue dendritic cells correlated with obesity-associated insulin-resistance and inducing Th17 responses in mice and patients. Diabetes.

[199] Macdougall C.E., Wood E.G., Loschko J., Scagliotti V., Cassidy F.C., Robinson M.E., Feldhahn N., Castellano L., Voisin M.-B., Marelli-Berg F. (2018). Visceral Adipose Tissue Immune Homeostasis Is Regulated by the Crosstalk between Adipocytes and Dendritic Cell Subsets. Cell Metab..

[200] Gregor M.F., Hotamisligil G.S. (2011). Inflammatory Mechanisms in Obesity. Annu. Rev. Immunol..

[201] Sears B., Perry M. (2015). The role of fatty acids in insulin resistance. Lipids Health Dis..

[202] Vessby B., Uusitupa M., Hermansen K., Riccardi G., Rivellese A.A., Tapsell L.C., Nälsén C., Berglund L., Louheranta A., Rasmussen B.M. (2001). Substituting dietary saturated for monounsaturated fat impairs insulin sensitivity in healthy men and women: The KANWU study. Diabetologia.

[203] Mayer-Davis E.J. (1998). Intensity and Amount of Physical Activity in Relation to Insulin Sensitivity: The Insulin Resistance Atherosclerosis Study. JAMA.

[204] Cefalu W.T. (2001). Insulin Resistance: Cellular and Clinical Concepts. Exp. Biol. Med..

[205] Uysal K.T., Wiesbrock S.M., Marino M.W., Hotamisligil G.S. (1997). Protection from obesity-induced insulin resistance in mice lacking TNF-α function. Nature.

[206] Yuan M. (2001). Reversal of Obesity- and Diet-Induced Insulin Resistance with Salicylates or Targeted Disruption of Ikkbeta. Science.

[207] Kim J.K., Kim Y.-J., Fillmore J.J., Chen Y., Moore I., Lee J., Yuan M., Li Z.W., Karin M., Perret P. (2001). Prevention of fat-induced insulin resistance by salicylate. J. Clin. Investig..

[208] Aguirre V., Uchida T., Yenush L., Davis R., White M.F. (2000). The c-Jun NH_2_-terminal Kinase Promotes Insulin Resistance during Association with Insulin Receptor Substrate-1 and Phosphorylation of Ser^307^*. J. Biol. Chem..

[209] Thaler J.P., Schwartz M.W. (2010). Minireview: Inflammation and Obesity Pathogenesis: The Hypothalamus Heats Up. Endocrinology.

[210] Velloso L.A., Schwartz M.W. (2011). Altered hypothalamic function in diet-induced obesity. Int. J. Obes..

[211] Valdearcos M., Robblee M.M., Benjamin D.I., Nomura D.K., Xu A.W., Koliwad S.K. (2014). Microglia Dictate the Impact of Saturated Fat Consumption on Hypothalamic Inflammation and Neuronal Function. Cell Rep..

[212] Spencer S.J., Tilbrook A. (2011). The glucocorticoid contribution to obesity. Stress.

[213] De Souza C.T., Araujo E.P., Bordin S., Ashimine R., Zollner R.L., Boschero A.C., Saad M.J.A., Velloso L.A. (2005). Consumption of a Fat-Rich Diet Activates a Proinflammatory Response and Induces Insulin Resistance in the Hypothalamus. Endocrinology.

[214] Posey K.A., Clegg D.J., Printz R.L., Byun J., Morton G.J., Vivekanandan-Giri A., Pennathur S., Baskin D.G., Heinecke J.W., Woods S.C. (2009). Hypothalamic proinflammatory lipid accumulation, inflammation, and insulin resistance in rats fed a high-fat diet. Am. J. Physiol.-Endocrinol. Metab..

[215] Jaworski K., Sarkadi-Nagy E., Duncan R.E., Ahmadian M., Sul H.S. (2007). Regulation of Triglyceride Metabolism.IV. Hormonal regulation of lipolysis in adipose tissue. Am. J. Physiol.-Gastrointest. Liver Physiol..

[216] Wilcox G. (2005). Insulin and insulin resistance. Clin. Biochem. Rev..

[217] Tokarz V.L., MacDonald P.E., Klip A. (2018). The cell biology of systemic insulin function. J. Cell Biol..

[218] Haeusler R.A., McGraw T.E., Accili D. (2018). Biochemical and cellular properties of insulin receptor signalling. Nat. Rev. Mol. Cell Biol..

[219] Olefsky J.M., Glass C.K. (2010). Macrophages, Inflammation, and Insulin Resistance. Annu. Rev. Physiol..

[220] Bachanek M., Abdalla N., Cendrowski K., Sawicki W. (2015). Value of ultrasonography in the diagnosis of polycystic ovary syndrome—literature review. J. Ultrason..

[221] Bachelot A. (2016). Polycystic ovarian syndrome: Clinical and biological diagnosis. Ann. Biol. Clin..

[222] Glintborg D., Andersen M. (2017). MANAGEMENT OF ENDOCRINE DISEASE: Morbidity in polycystic ovary syndrome. Eur. J. Endocrinol..

[223] Pourghassem Gargari B., Houjeghani S., Farzadi L., Houjeghani S., Safaeiyan A. (2015). Relationship between Serum Leptin, Ghrelin and Dietary Macronutrients in Women with Polycystic Ovary Syndrome. Int. J. Fertil. Steril..

[224] Jalilian A., Kiani F., Sayehmiri F., Sayehmiri K., Khodaee Z., Akbari M. (2015). Prevalence of polycystic ovary syndrome and its associated complications in Iranian women: A meta-analysis. Iran. J. Reprod. Med..

[225] Carvalho L.M.L., Dos Reis F.M., Candido A.L., Nunes F.F.C., Ferreira C.N., Gomes K.B. (2018). Polycystic Ovary Syndrome as a systemic disease with multiple molecular pathways: A narrative review. Endocr. Regul..

[226] Jacobs H.S., Conway G.S. (1999). Leptin, polycystic ovaries and polycystic ovary syndrome. Hum. Reprod. Update.

[227] Moran L., Brinkworth G., Norman R. (2008). Dietary Therapy in Polycystic Ovary Syndrome. Semin. Reprod. Med..

[228] Faghfoori Z., Fazelian S., Shadnoush M., Goodarzi R. (2017). Nutritional management in women with polycystic ovary syndrome: A review study. Diabetes Metab. Syndr. Clin. Res. Rev..

[229] Jena D., Choudhury A.K., Mangaraj S., Singh M., Mohanty B.K., Baliarsinha A.K. (2018). Study of Visceral and Subcutaneous Abdominal Fat Thickness and Its Correlation with Cardiometabolic Risk Factors and Hormonal Parameters in Polycystic Ovary Syndrome. Indian J. Endocrinol. Metab..

[230] Frøssing S., Nylander M.C., Chabanova E., Kistorp C., Skouby S.O., Faber J. (2018). Quantification of visceral adipose tissue in polycystic ovary syndrome: Dual-energy X-ray absorptiometry versus magnetic resonance imaging. Acta Radiol..

[231] Fain J.N., Bahouth S.W., Madan A.K. (2004). TNFα release by the nonfat cells of human adipose tissue. Int. J. Obes..

[232] Duleba A.J., Dokras A. (2012). Is PCOS an inflammatory process?. Fertil. Steril..

[233] Mortada R., Kallail K.J., Dong F., Karakas S. (2015). HbA1c in Patients with Polycystic Ovary Syndrome: A Potential Marker of Inflammation. J. Reprod. Infertil..

[234] Nehir Aytan A., Bastu E., Demiral I., Bulut H., Dogan M., Buyru F. (2016). Relationship between hyperandrogenism, obesity, inflammation and polycystic ovary syndrome. Gynecol. Endocrinol..

[235] Faubert J., Battista M.-C., Baillargeon J.-P. (2016). PHYSIOLOGY AND ENDOCRINOLOGY SYMPOSIUM: Insulin action and lipotoxicity in the development of polycystic ovary syndrome: A review1. J. Anim. Sci..

[236] Dunaif A. (2016). Perspectives in Polycystic Ovary Syndrome: From Hair to Eternity. J. Clin. Endocrinol. Metab..

[237] Kahsar-Miller M., Azziz R. (1998). The development of the polycystic ovary syndrome: Family history as a risk factor. Trends Endocrinol. Metab..

[238] Vink J.M., Sadrzadeh S., Lambalk C.B., Boomsma D.I. (2006). Heritability of Polycystic Ovary Syndrome in a Dutch Twin-Family Study. J. Clin. Endocrinol. Metab..

[239] Jia H., Yu L., Guo X., Gao W., Jiang Z. (2012). Associations of adiponectin gene polymorphisms with polycystic ovary syndrome: A meta-analysis. Endocrine.

[240] Ruan Y., Ma J., Xie X. (2012). Association of IRS-1 and IRS-2 genes polymorphisms with polycystic ovary syndrome: A meta-analysis. Endocr. J..

[241] Reddy T.V., Govatati S., Deenadayal M., Shivaji S., Bhanoori M. (2018). Polymorphisms in the TFAM and PGC1-α genes and their association with polycystic ovary syndrome among South Indian women. Gene.

[242] Sales M., Sóter M., Candido A., Fernandes A., Oliveira F., Ferreira A., Sousa M., Ferreira C., Gomes K. (2013). Correlation between plasminogen activator inhibitor-1 (PAI-1) promoter 4G/5G polymorphism and metabolic/proinflammatory factors in polycystic ovary syndrome. Gynecol. Endocrinol..

[243] Zhao H., Lv Y., Li L., Chen Z.-J. (2016). Genetic Studies on Polycystic Ovary Syndrome. Best Pract. Res. Clin. Obstet. Gynaecol..

[244] Hosseini A.H., Kohan L., Aledavood A., Rostami S. (2017). Association of miR-146a rs2910164 and miR-222 rs2858060 polymorphisms with the risk of polycystic ovary syndrome in Iranian women: A case–control study. Taiwan. J. Obstet. Gynecol..

[245] Korhonen S. (2003). Polymorphism in the peroxisome proliferator-activated receptor-gamma gene in women with polycystic ovary syndrome. Hum. Reprod..

[246] Yilmaz M., Ergün M.A., Karakoç A., Yurtçu E., Yetkin İ., Ayvaz G., Çakir N., Arslan M. (2005). Pro12Ala polymorphism of the *peroxisome proliferator-activated receptor-γ* gene in first-degree relatives of subjects with polycystic ovary syndrome. Gynecol. Endocrinol..

[247] Yilmaz M., Ali Ergün M., Karakoç A., Yurtçu E., Çakir N., Arslan M. (2006). Pro12Ala polymorphism of the peroxisome proliferator-activated receptor-γ gene in women with polycystic ovary syndrome. Gynecol. Endocrinol..

[248] Orio F., Matarese G., Di Biase S., Palomba S., Labella D., Sanna V., Savastano S., Zullo F., Colao A., Lombardi G. (2003). Exon 6 and 2 Peroxisome Proliferator-Activated Receptor-γ Polymorphisms in Polycystic Ovary Syndrome. J. Clin. Endocrinol. Metab..

[249] Orio F., Palomba S., Cascella T., Di Biase S., Labella D., Russo T., Savastano S., Zullo F., Colao A., Vettor R. (2004). Lack of an Association between Peroxisome Proliferator-Activated Receptor-γ Gene Pro12Ala Polymorphism and Adiponectin Levels in the Polycystic Ovary Syndrome. J. Clin. Endocrinol. Metab..

[250] Hahn S., Fingerhut A., Khomtsiv U., Khomtsiv L., Tan S., Quadbeck B., Herrmann B.L., Knebel B., Muller-Wieland D., Mann K. (2005). The peroxisome proliferator activated receptor gamma Pro12Ala polymorphism is associated with a lower hirsutism score and increased insulin sensitivity in women with polycystic ovary syndrome. Clin. Endocrinol..

[251] Skov V., Glintborg D., Knudsen S., Tan Q., Jensen T., Kruse T.A., Beck-Nielsen H., Højlund K. (2008). Pioglitazone enhances mitochondrial biogenesis and ribosomal protein biosynthesis in skeletal muscle in polycystic ovary syndrome. PLoS ONE.

[252] Buse J.B., Tan M.H., Prince M.J., Erickson P.P. (2004). The effects of oral anti-hyperglycaemic medications on serum lipid profiles in patients with type 2 diabetes. Diabetes Obes. Metab..

[253] Lewis G.F., Carpentier A., Adeli K., Giacca A. (2002). Disordered Fat Storage and Mobilization in the Pathogenesis of Insulin Resistance and Type 2 Diabetes. Endocr. Rev..

[254] Norman R.J., Dewailly D., Legro R.S., Hickey T.E. (2007). Polycystic ovary syndrome. Lancet.

[255] Al-Eisa E., Gabr S.A., Alghadir A.H. (2017). Effects of supervised aerobic training on the levels of anti-Mullerian hormone and adiposity measures in women with normo-ovulatory and polycystic ovary syndrome. J. Pak. Med. Assoc..

[256] Palioura E., Diamanti-Kandarakis E. (2013). Industrial endocrine disruptors and polycystic ovary syndrome. J. Endocrinol. Investig..

[257] Varlamov O., Bishop C.V., Handu M., Takahashi D., Srinivasan S., White A., Roberts C.T. (2017). Combined androgen excess and Western-style diet accelerates adipose tissue dysfunction in young adult, female nonhuman primates. Hum. Reprod..

[258] Rizkalla S.W., Taghrid L., Laromiguiere M., Huet D., Boillot J., Rigoir A., Elgrably F., Slama G. (2004). Improved plasma glucose control, whole-body glucose utilization, and lipid profile on a low-glycemic index diet in type 2 diabetic men: A randomized controlled trial. Diabetes Care.

[259] Ebbeling C.B., Leidig M.M., Sinclair K.B., Seger-Shippee L.G., Feldman H.A., Ludwig D.S. (2005). Effects of an ad libitum low-glycemic load diet on cardiovascular disease risk factors in obese young adults. Am. J. Clin. Nutr..

[260] Haqq L., McFarlane J., Dieberg G., Smart N. (2014). Effect of lifestyle intervention on the reproductive endocrine profile in women with polycystic ovarian syndrome: A systematic review and meta-analysis. Endocr. Connect..

[261] Teede H.J., Misso M.L., Costello M.F., Dokras A., Laven J., Moran L., Piltonen T., Norman R.J., International PCOS Network (2018). Recommendations from the international evidence-based guideline for the assessment and management of polycystic ovary syndrome†‡. Hum. Reprod..

[262] Valsamakis G., Lois K., Kumar S., Mastorakos G. (2013). Metabolic and other effects of pioglitazone as an add-on therapy to metformin in the treatment of polycystic ovary syndrome (PCOS). Hormones.

[263] Froment P., Touraine P. (2006). Thiazolidinediones and Fertility in Polycystic Ovary Syndrome (PCOS). PPAR Res..

[264] Baillargeon J.-P., Jakubowicz D.J., Iuorno M.J., Jakubowicz S., Nestler J.E. (2004). Effects of metformin and rosiglitazone, alone and in combination, in nonobese women with polycystic ovary syndrome and normal indices of insulin sensitivity. Fertil. Steril..

[265] Veldhuis J.D., Zhang G., Garmey J.C. (2002). Troglitazone, an insulin-sensitizing thiazolidinedione, represses combined stimulation by LH and insulin of de novo androgen biosynthesis by thecal cells in vitro. J. Clin. Endocrinol. Metab..

[266] Kempná P., Hofer G., Mullis P.E., Flück C.E. (2007). Pioglitazone inhibits androgen production in NCI-H295R cells by regulating gene expression of CYP17 and HSD3B2. Mol. Pharm..

[267] Li M., Pan L.C., Simmons H.A., Li Y., Healy D.R., Robinson B.S., Ke H.Z., Brown T.A. (2006). Surface-specific effects of a PPARgamma agonist, darglitazone, on bone in mice. Bone.

[268] Abbas A., Blandon J., Rude J., Elfar A., Mukherjee D. (2012). PPAR-γ agonist in treatment of diabetes: Cardiovascular safety considerations. Cardiovasc. Hematol. Agents Med. Chem..

[269] Vernon G., Baranova A., Younossi Z.M. (2011). Systematic review: The epidemiology and natural history of non-alcoholic fatty liver disease and non-alcoholic steatohepatitis in adults: Systematic review: Epidemiology of NAFLD and NASH. Aliment. Pharmacol. Ther..

[270] Thoma C., Day C.P., Trenell M.I. (2012). Lifestyle interventions for the treatment of non-alcoholic fatty liver disease in adults: A systematic review. J. Hepatol..

[271] Sayiner M., Koenig A., Henry L., Younossi Z.M. (2016). Epidemiology of Nonalcoholic Fatty Liver Disease and Nonalcoholic Steatohepatitis in the United States and the Rest of the World. Clin. Liver Dis..

[272] Kanwar P., Kowdley K.V. (2016). The Metabolic Syndrome and Its Influence on Nonalcoholic Steatohepatitis. Clin. Liver Dis..

[273] Brunt E.M., Tiniakos D.G. (2010). Histopathology of nonalcoholic fatty liver disease. World J. Gastroenterol..

[274] Bugianesi E., McCullough A.J., Marchesini G. (2005). Insulin resistance: A metabolic pathway to chronic liver disease. Hepatology.

[275] Chao H.-W., Chao S.-W., Lin H., Ku H.-C., Cheng C.-F. (2019). Homeostasis of Glucose and Lipid in Non-Alcoholic Fatty Liver Disease. Int. J. Mol. Sci..

[276] Dowman J.K., Tomlinson J.W., Newsome P.N. (2010). Pathogenesis of non-alcoholic fatty liver disease. QJM.

[277] Day C.P., James O.F.W. (1998). Steatohepatitis: A tale of two “hits”?. Gastroenterology.

[278] Masarone M., Rosato V., Dallio M., Gravina A.G., Aglitti A., Loguercio C., Federico A., Persico M. (2018). Role of Oxidative Stress in Pathophysiology of Nonalcoholic Fatty Liver Disease. Oxid. Med. Cell. Longev..

[279] Ucar F., Sezer S., Erdogan S., Akyol S., Armutcu F., Akyol O. (2013). The relationship between oxidative stress and nonalcoholic fatty liver disease: Its effects on the development of nonalcoholic steatohepatitis. Redox Rep..

[280] Souza-Mello V., Gregório B.M., Cardoso-de-Lemos F.S., de Carvalho L., Aguila M.B., Mandarim-de-Lacerda C.A. (2010). Comparative effects of telmisartan, sitagliptin and metformin alone or in combination on obesity, insulin resistance, and liver and pancreas remodelling in C57BL/6 mice fed on a very high-fat diet. Clin. Sci..

[281] Doege H., Baillie R.A., Ortegon A.M., Tsang B., Wu Q., Punreddy S., Hirsch D., Watson N., Gimeno R.E., Stahl A. (2006). Targeted Deletion of FATP5 Reveals Multiple Functions in Liver Metabolism: Alterations in Hepatic Lipid Homeostasis. Gastroenterology.

[282] Falcon A., Doege H., Fluitt A., Tsang B., Watson N., Kay M.A., Stahl A. (2010). FATP2 is a hepatic fatty acid transporter and peroxisomal very long-chain acyl-CoA synthetase. Am. J. Physiol.-Endocrinol. Metab..

[283] Sookoian S., Pirola C.J. (2017). Genetic predisposition in nonalcoholic fatty liver disease. Clin. Mol. Hepatol..

[284] Donnelly K.L., Smith C.I., Schwarzenberg S.J., Jessurun J., Boldt M.D., Parks E.J. (2005). Sources of fatty acids stored in liver and secreted via lipoproteins in patients with nonalcoholic fatty liver disease. J. Clin. Investig..

[285] Petersen K.F., Dufour S., Savage D.B., Bilz S., Solomon G., Yonemitsu S., Cline G.W., Befroy D., Zemany L., Kahn B.B. (2007). The role of skeletal muscle insulin resistance in the pathogenesis of the metabolic syndrome. Proc. Natl. Acad. Sci. USA.

[286] Motomura W., Inoue M., Ohtake T., Takahashi N., Nagamine M., Tanno S., Kohgo Y., Okumura T. (2006). Up-regulation of ADRP in fatty liver in human and liver steatosis in mice fed with high fat diet. Biochem. Biophys. Res. Commun..

[287] Schadinger S.E., Bucher N.L.R., Schreiber B.M., Farmer S.R. (2005). PPARγ2 regulates lipogenesis and lipid accumulation in steatotic hepatocytes. Am. J. Physiol.-Endocrinol. Metab..

[288] Wijarnpreecha K., Thongprayoon C., Panjawatanan P., Ungprasert P. (2016). Short sleep duration and risk of nonalcoholic fatty liver disease: A systematic review and meta-analysis: Coffee and nonalcoholic fatty liver disease. J. Gastroenterol. Hepatol..

[289] Santaliestra-Pasías A.M., Mouratidou T., Huybrechts I., Beghin L., Cuenca-García M., Castillo M.J., Galfo M., Hallstrom L., Kafatos A., Manios Y. (2014). Increased sedentary behaviour is associated with unhealthy dietary patterns in European adolescents participating in the HELENA study. Eur. J. Clin. Nutr..

[290] Younossi Z.M., Koenig A.B., Abdelatif D., Fazel Y., Henry L., Wymer M. (2016). Global epidemiology of nonalcoholic fatty liver disease-Meta-analytic assessment of prevalence, incidence, and outcomes. Hepatology.

[291] Farrell G.C., Wong V.W.-S., Chitturi S. (2013). NAFLD in Asia—as common and important as in the West. Nat. Rev. Gastroenterol. Hepatol..

[292] Perumpail B.J., Cholankeril R., Yoo E.R., Kim D., Ahmed A. (2017). An Overview of Dietary Interventions and Strategies to Optimize the Management of Non-Alcoholic Fatty Liver Disease. Diseases.

[293] Mosca A., Nobili V., De Vito R., Crudele A., Scorletti E., Villani A., Alisi A., Byrne C.D. (2017). Serum uric acid concentrations and fructose consumption are independently associated with NASH in children and adolescents. J. Hepatol..

[294] Bawden S., Stephenson M., Falcone Y., Lingaya M., Ciampi E., Hunter K., Bligh F., Schirra J., Taylor M., Morris P. (2017). Increased liver fat and glycogen stores after consumption of high versus low glycaemic index food: A randomized crossover study: BAWDEN et al. Diabetes Obes. Metab..

[295] Mager D.R., Iñiguez I.R., Gilmour S., Yap J. (2015). The Effect of a Low Fructose and Low Glycemic Index/Load (FRAGILE) Dietary Intervention on Indices of Liver Function, Cardiometabolic Risk Factors, and Body Composition in Children and Adolescents With Nonalcoholic Fatty Liver Disease (NAFLD). J. Parenter. Enter. Nutr..

[296] Misciagna G., del Pilar Díaz M., Caramia D.V., Bonfiglio C., Franco I., Noviello M.R., Chiloiro M., Abbrescia D.I., Mirizzi A., Tanzi M. (2017). Effect of a low glycemic index Mediterranean diet on non-alcoholic fatty liver disease. A randomized controlled clinici trial. J. Nutr. Health Aging.

[297] Hashida R., Kawaguchi T., Bekki M., Omoto M., Matsuse H., Nago T., Takano Y., Ueno T., Koga H., George J. (2017). Aerobic vs. resistance exercise in non-alcoholic fatty liver disease: A systematic review. J. Hepatol..

[298] Koltyn K.F., Brellenthin A.G., Cook D.B., Sehgal N., Hillard C. (2014). Mechanisms of Exercise-Induced Hypoalgesia. J. Pain.

[299] Tutunchi H., Ostadrahimi A., Saghafi-Asl M., Maleki V. (2019). The effects of oleoylethanolamide, an endogenous PPAR-α agonist, on risk factors for NAFLD: A systematic review. Obes. Rev..

[300] Lu W., Li S., Li J., Wang J., Zhang R., Zhou Y., Yin Q., Zheng Y., Wang F., Xia Y. (2016). Effects of Omega-3 Fatty Acid in Nonalcoholic Fatty Liver Disease: A Meta-Analysis. Gastroenterol. Res. Pract..

[301] Bellentani S., Dalle Grave R., Suppini A., Marchesini G. (2007). Behavior therapy for nonalcoholic fatty liver disease: The need for a multidisciplinary approach. Hepatology.

[302] McCarthy E.M., Rinella M.E. (2012). The Role of Diet and Nutrient Composition in Nonalcoholic Fatty Liver Disease. J. Acad. Nutr. Diet..

[303] Mouzaki M., Allard J.P. (2012). The Role of Nutrients in the Development, Progression, and Treatment of Nonalcoholic Fatty Liver Disease. J. Clin. Gastroenterol..

[304] Ioannou G.N. (2016). The Role of Cholesterol in the Pathogenesis of NASH. Trends Endocrinol. Metab..

[305] Kersten S., Desvergne B., Wahli W. (2000). Roles of PPARs in health and disease. Nature.

[306] Clarke S.D.I. (2001). Molecular mechanism for polyunsaturated fatty acid regulation of gene transcription. Am. J. Physiol.-Gastrointest. Liver Physiol..

[307] Jump D.B., Botolin D., Wang Y., Xu J., Christian B., Demeure O. (2005). Fatty Acid Regulation of Hepatic Gene Transcription. J. Nutr..

[308] Lombardo Y.B., Chicco A.G. (2006). Effects of dietary polyunsaturated n-3 fatty acids on dyslipidemia and insulin resistance in rodents and humans. A review. J. Nutr. Biochem..

[309] Pachikian B.D., Essaghir A., Demoulin J.-B., Neyrinck A.M., Catry E., De Backer F.C., Dejeans N., Dewulf E.M., Sohet F.M., Portois L. (2011). Hepatic n-3 Polyunsaturated Fatty Acid Depletion Promotes Steatosis and Insulin Resistance in Mice: Genomic Analysis of Cellular Targets. PLoS ONE.

[310] George J., Liddle C. (2008). Nonalcoholic Fatty Liver Disease: Pathogenesis and Potential for Nuclear Receptors as Therapeutic Targets. Mol. Pharm..

[311] Fraulob J.C., Souza-Mello V., Aguila M.B., Mandarim-de-Lacerda C.A. (2012). Beneficial effects of rosuvastatin on insulin resistance, adiposity, inflammatory markers and non-alcoholic fatty liver disease in mice fed on a high-fat diet. Clin. Sci..

[312] Shapiro H., Tehilla M., Attal-Singer J., Bruck R., Luzzatti R., Singer P. (2011). The therapeutic potential of long-chain omega-3 fatty acids in nonalcoholic fatty liver disease. Clin. Nutr..

[313] Ruiz-Núñez B., Pruimboom L., Dijck-Brouwer D.A.J., Muskiet F.A.J. (2013). Lifestyle and nutritional imbalances associated with Western diseases: Causes and consequences of chronic systemic low-grade inflammation in an evolutionary context. J. Nutr. Biochem..

[314] Giampietro L., Laghezza A., Cerchia C., Florio R., Recinella L., Capone F., Ammazzalorso A., Bruno I., De Filippis B., Fantacuzzi M. (2019). Novel Phenyldiazenyl Fibrate Analogues as PPAR α/γ/δ Pan-Agonists for the Amelioration of Metabolic Syndrome. ACS Med. Chem. Lett..

